# Increased fibroblast growth factor-21 in chronic kidney disease is a trade-off between survival benefit and blood pressure dysregulation

**DOI:** 10.1038/s41598-019-55643-4

**Published:** 2019-12-17

**Authors:** Toshihiro Nakano, Kazuhiro Shiizaki, Yutaka Miura, Masahiro Matsui, Keisei Kosaki, Shoya Mori, Kunihiro Yamagata, Seiji Maeda, Takuya Kishi, Naoki Usui, Masahide Yoshida, Tatsushi Onaka, Hiroaki Mizukami, Ruri Kaneda, Kazunori Karasawa, Kosaku Nitta, Hiroshi Kurosu, Makoto Kuro-o

**Affiliations:** 1 Division of Anti-aging Medicine, Center for Molecular Medicine, Jichi Medical University, Tochigi, Japan; 2 Department of Medicine IV, Tokyo Women’s Medical University, Tokyo, Japan; 3 Graduate School of Comprehensive Human Sciences, University of Tsukuba, Ibaraki, Japan; 4 Faculty of Sport Sciences, Waseda University, Saitama, Japan; 5 Faculty of Health and Sport Sciences, University of Tsukuba, Ibaraki, Japan; 6 Japan Society for the Promotion of Science, Tokyo, Japan; 7 Department of Nephrology, Faculty of Medicine, University of Tsukuba, Ibaraki, Japan; 8 Faculty of Health and Welfare Sciences in Fukuoka, International University of Health and Welfare, Fukuoka, Japan; 9 Division of Brain and Neurophysiology, Department of Physiology, Jichi Medical University, Tochigi, Japan; 10 Division of Genetic Therapeutics, Center for Molecular Medicine, Jichi Medical University, Tochigi, Japan; 11 Charles and Jane Pak Center for Mineral Metabolism and Clinical Research, Department of Internal Medicine, University of Texas Southwestern Medical Center, Dallas, Texas USA; 12 AMED-CREST, Japan Agency for Medical Research and Development, Tokyo, Japan

**Keywords:** Endocrinology, Nephrology

## Abstract

Circulating levels of fibroblast growth factor-21 (FGF21) start increasing in patients with chronic kidney disease (CKD) since early stages during the cause of disease progression. FGF21 is a liver-derived hormone that induces responses to stress through acting on hypothalamus to activate the sympathetic nervous system and the hypothalamus-pituitary-adrenal endocrine axis. However, roles that FGF21 plays in pathophysiology of CKD remains elusive. Here we show in mice that FGF21 is required to survive CKD but responsible for blood pressure dysregulation. When introduced with CKD, *Fgf21*^−/−^ mice died earlier than wild-type mice. Paradoxically, these *Fgf21*^−/−^ CKD mice escaped several complications observed in wild-type mice, including augmentation of blood pressure elevating response and activation of the sympathetic nervous system during physical activity and increase in serum noradrenalin and corticosterone levels. Supplementation of FGF21 by administration of an FGF21-expressing adeno-associated virus vector recapitulated these complications in wild-type mice and restored the survival period in *Fgf21*^−/−^ CKD mice. In CKD patients, high serum FGF21 levels are independently associated with decreased baroreceptor sensitivity. Thus, increased FGF21 in CKD can be viewed as a survival response at the sacrifice of blood pressure homeostasis.

## Introduction

Three members of the fibroblast growth factor (FGF) family, FGF19 (the human ortholog of rodent FGF15), FGF21, and FGF23, are distinct from the other FGF family members in two major aspects. First, unlike the other FGFs that function as paracrine/autocrine growth factors, these three FGFs function as hormones that regulate various metabolic processes, including mineral, glucose, fatty acid, and bile acid metabolism^1^. Hence, these three FGFs are collectively called endocrine FGFs. Second, endocrine FGFs require the Klotho family of type-I transmembrane proteins (either αKlotho or βKlotho) for high-affinity binding to their cognate FGF receptor (FGFR) tyrosine kinases^2–4^. Klotho proteins form binary complexes with specific FGFR isoforms to create an extracellular groove between Klotho and FGFR, in which endocrine FGFs fit^5,6^. Formation of the Klotho-FGFR-FGF ternary complex is required for activation of the canonical FGF signaling pathway^5^. Accordingly, target organs of endocrine FGFs are determined by expression profile of the Klotho and FGFR isoforms^7^. Specifically, the major target organ of FGF19 is the liver where the βKlotho-FGFR4 complex is expressed^4^. Likewise, FGF21 acts on adipocytes^3^ and neurons in the suprachiasmatic nucleus (SCN)^8^ where βKlotho and FGFR1c are co-expressed. FGF23 binds to the αKlotho complexed with either FGFR1c, FGFR3c, or FGFR4 expressed on renal tubular cells^2^.

Chronic kidney disease (CKD) is defined as any kidney damage that persists beyond three months^9^. In many cases, CKD occurs as a complication of hypertension and diabetes mellitus and/or as a result of the natural course of aging. Thus, CKD is one of the most prevalent diseases in industrialized countries, affecting more than 10% of the total population^10^. Regardless of the cause of kidney damage, the common pathology of CKD is considered decrease in the functional nephron number^1^.

Numerous studies have demonstrated that circulating FGF23 levels are increased during CKD progression^11^. FGF23 is secreted from the bone (osteoblasts and osteocytes) in response to dietary phosphate intake. FGF23 then acts on the kidney to increase urinary phosphate excretion through suppressing phosphate reabsorption at renal tubules^12^, thereby maintaining the phosphate balance. Because FGF23 increases phosphate excretion per nephron, it is conceivable that the progressive increase in circulating FGF23 levels as CKD advances compensates for the progressive decrease in the functional nephron number to maintain phosphate homeostasis^1^.

There are a few clinical studies showing that not only FGF23 but also FGF21 are increased during CKD progression^13,14^. FGF21 is secreted from hepatocytes in response to various types of stress, including fasting^15^ and inflammation^16^. FGF21 crosses the blood brain barrier, enter the central nervous system, and acts on the SCN^8^. FGF21 then induces expression of corticotropin releasing hormone (CRH) through a neural pathway(s) yet to be determined. CRH activates the hypothalamus-pituitary-adrenal (HPA) axis and the sympathetic nervous system, thereby inducing responses to stress^8^. Interestingly, transgenic mice that overexpress FGF21 live longer than wild-type mice^17^, which is consistent with an evolutionarily conserved law that increased ability to handle with stress is associated with extended longevity^18^.

Therefore, we hypothesize that the progressive increase in FGF21 as CKD advances may be a survival response to cope with growing stress caused by CKD progression. To test this hypothesis, we introduced CKD into mice lacking FGF21 to determine whether their prognosis might be poorer than wild-type mice.

## Results

### FGF21 is required to survive CKD

**Figure 1:**
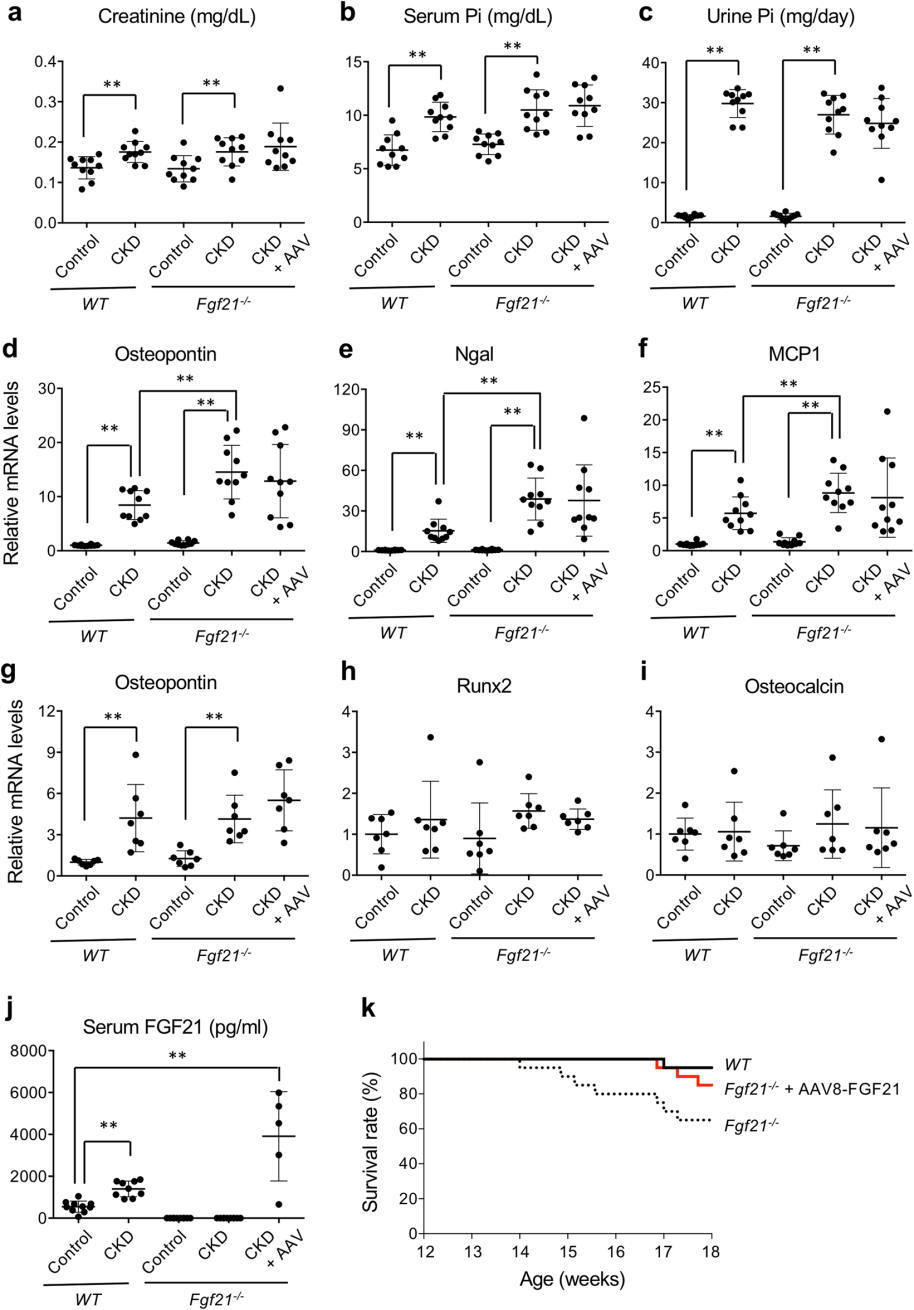
FGF21 is required to survive CKD. Wild-type mice (*WT*) and FGF21 knockout mice (*Fgf21*^−/−^) were subjected to either uninephrectomy followed by high phosphate diet feeding (CKD) or sham-operation followed by normal diet feeding (Control). Ten out of the 20 *Fgf21*^−/−^ CKD mice were administered with the AAV8-FGF21 vector when the high phosphate diet feeding was started at 12 weeks of age (CKD + AAV). Serum creatinine (**a**), serum phosphate (**b**), and urine phosphate (**c**) were indicated. Relative mRNA expression levels of osteopontin (**d**), Ngal (**e**), and MCP1 (**f**) in the kidney were indicated. Relative mRNA expression levels of osteopontin (**g**), Runx2 (**h**), and osteocalcin (**i**) in the aorta were indicated. (**j**) Serum FGF21 levels. The bars indicate mean ± SD, **P < 0.01, *P < 0.05 by t-test. (**k**) Survival curves of CKD mice. FGF21 knockout mice (*Fgf21*^−/−^) showed poorer prognosis than wild-type mice (WT, P = 0.017 by log-rank test), but survived as well as WT when treated with the AAV8-FGF21 vector at 12 weeks of age.

*Fgf21*^−/−^ mice and wild-type mice were uninephrectomized and then placed on high phosphate diet for 5 weeks to introduce chronic kidney damage^19^. As controls for these CKD model mice, *Fgf21*^−/−^ mice and wild-type mice were sham-operated (laparotomy alone) and placed on normal diet. None of these control mice died before the end of the observation period when the mice reached 18 weeks of age. The CKD mice exhibited modest increase in serum creatinine (Fig. 1a) and phosphate (Fig. 1b) levels. There was no difference in the amount of dietary phosphate load between wild-type CKD mice and *Fgf21*^−/−^ CKD mice, because daily urinary phosphate excretion was comparable between them (Fig. 1c). However, *Fgf21*^−/−^ CKD mice exhibited severer renal tubular damage and inflammation than wild-type CKD mice as determined by expression levels of osteopontin, neutrophil gelatinase-associated lipocalin (Ngal), and monocyte chemotactic protein-1 (MCP1) (Fig. 1d–f). Although vascular calcification was undetectable in these CKD mice at the histological level, expression levels of markers for vascular calcification such as osteopontin, but not runt-related transcription factor-2 (Runx2) and osteocalcin, were increased in aorta both in wild-type CKD mice and *Fgf21*^−/−^ CKD mice to the similar extent (Fig. 1g–i).

The wild-type CKD mice had higher circulating FGF21 levels than the wild-type control mice (Fig. 1j). The Kaplan-Meier analysis showed that the survival rate of *Fgf21*^−/−^ CKD mice was lower than that of wild-type CKD mice (Fig. 1k). Although *Fgf21*^−/−^ CKD mice developed severer kidney damage than wild-type CKD mice (Fig. 1d–f), this did not explain the poor prognosis of *Fgf21*^−/−^ CKD mice, because restoration of their circulating FGF21 levels by administration of an FGF21-expressing adeno-associated virus vector (AAV8-FGF21) improved their survival (Fig. 1k) without improving the kidney damage (Fig. 1d–f). These findings indicate that the kidney damage *per se* was not the cause of death in *Fgf21*^−/−^ CKD mice. Rather, their poor prognosis was due to an unidentified extrarenal complication(s) manifested by loss of FGF21. Although we were unable to specify the cause of death in *Fgf21*^−/−^ CKD mice in this study, we can safely conclude that FGF21 is indispensable to survive CKD.

### Blood pressure dysregulation in CKD mice

Although transgenic overexpression of FGF21 was reported to extend life span in mice^17^, it also led to some adverse outcomes including disturbed circadian behavior through the excess FGF21 acting on the SCN, the center for circadian rhythm regulation^8^. Therefore, we speculated that the increase in circulating FGF21 levels in CKD, while indispensable for survival, might be responsible for some CKD complications related to circadian rhythm dysregulation.

**Figure 2:**
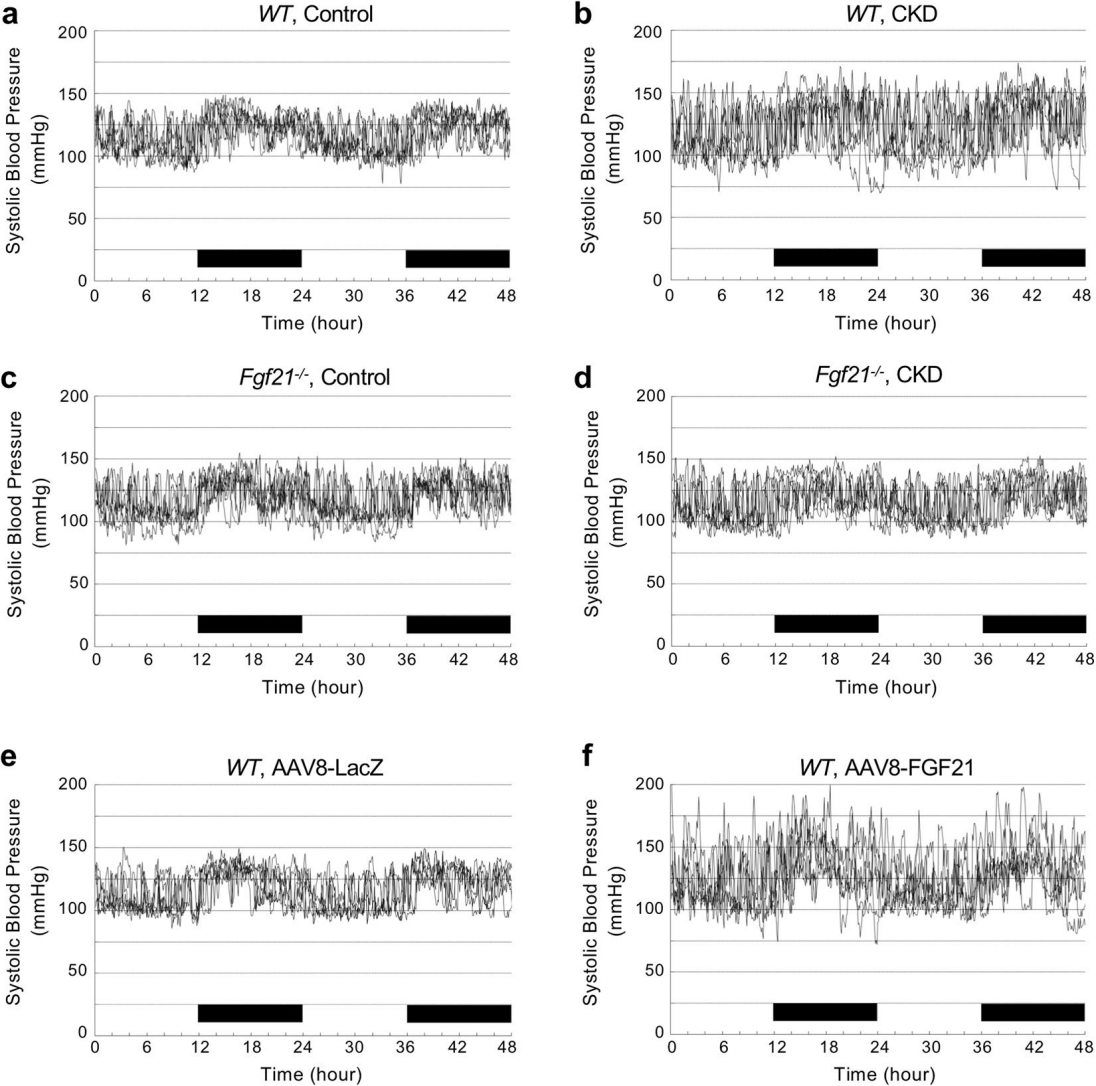
Effects of FGF21 on blood pressure. Arterial pressure was monitored continuously for 48 hours using a mouse telemetry system in wild-type control mice (**a**), wild-type CKD mice (**b**), *Fgf21*^−/−^ control mice (**c**), *Fgf21*^−/−^ CKD mice (**d**), wild-type mice injected with the AAV8-LacZ (**e**), and wild-type mice injected with the AAV8-FGF21 (**f**). The systolic blood pressure data from 6 mice were overlapped in each group. The wild-type CKD mice (**b**) and wild-type mice injected with the AAV8-FGF21 (**f**) showed higher variability of systolic blood pressure than the other mice. The black bars indicate the nighttime (light off).

In healthy individuals, the blood pressure falls while sleeping and rises while waking up. In CKD patients, however, this normal circadian rhythm of blood pressure is frequently perturbed^20^. Thus, we hypothesized that FGF21 might be responsible for the disturbed circadian rhythm of blood pressure in CKD. To test this hypothesis in the mouse CKD model, we placed a catheter in the internal carotid artery and measured arterial blood pressure continuously over for 48 hours under the conscious and unrestricted condition using a telemetry system. Contrary to our expectation, wild-type CKD mice exhibited the circadian rhythm of blood pressure, although less obvious than that seen in wild-type control mice (Fig. 2a,b). Similarly, *Fgf21*^−/−^ control mice and *Fgf21*^−/−^ CKD mice exhibited the circadian rhythm of blood pressure indistinguishable from that of wild-type control mice (Fig. 2c,d). FGF21 appears to have little effect on the diurnal rhythm of blood pressure.

**Figure 3:**
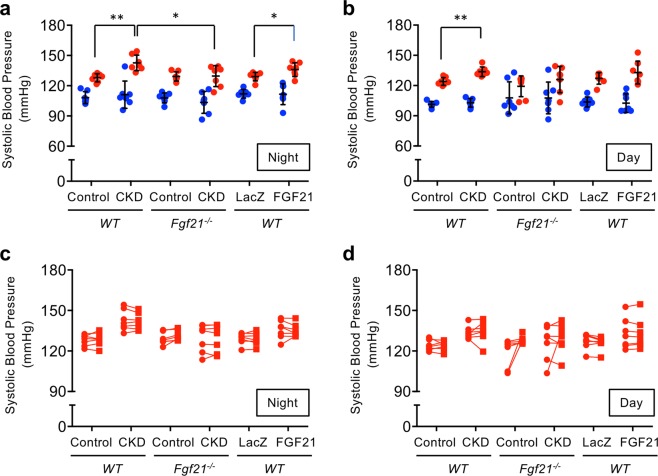
FGF21 augments the pressor response during physical activity. The histograms of systolic blood pressure (SBP) were modeled as sum of two normal distributions, Distribution 1 (D1) and Distribution 2 (D2). (**a**) The averages of D1 (blue circle) and D2 (red circle) in the nighttime. The averages of D1 were similar across the groups. The averages of D2 in the wild-type CKD mice were significantly higher than those in the wild-type control mice. The averages of D2 in the *Fgf21*^−/−^ CKD mice were not different from those in the *Fgf21*^−/−^ control mice and the wild-type control mice and significantly lower than those of the wild-type CKD mice. The averages of D2 in the wild-type mice treated with the AAV8-FGF21 vector were significantly higher than those in the wild-type mice treated with the control vector (LacZ) and similar to those in the wild-type CKD mice. (**b**) The averages of D1 (blue circle) and D2 (red circle) in the daytime. As in the nighttime, the averages of D2 in the wild-type CKD mice were significantly higher than those in the wild-type control mice. However, the difference between the wild-type CKD mice and the *Fgf21*^−/−^ CKD mice did not reach statistical significance. The difference between the wild-type mice treated with the AAV8-FGF21 vector and the wild-type mice treated with the control vector did not reach statistical significance, either. The bars indicate mean ± SD, **P < 0.01, *P < 0.05 by t-test. (**c**, **d**) The averages of D2 (red circle) were similar to the averages of SBP during physical activity (red square).

Instead, we noticed that the blood pressure of the wild-type CKD mice fluctuated more robustly than that of the wild-type control mice (Fig. 2a,b). To explore the cause of the increased blood pressure variability induced by CKD, we analyzed histograms of systolic blood pressure in individual mice (Supplementary Figs. S1–S12). The histograms showed bimodal distribution that were modeled as sum of two normal distributions with the lower and the higher average, which we designated as Distribution 1 (D1) and Distribution 2 (D2), respectively. Although the mean of the D1 averages were not different between the groups, the mean of the D2 averages in the wild-type CKD mice was significantly higher than that of the wild-type control mice both in the nighttime and the daytime (Fig. 3a,b). The standard variations of the D1 averages and the D2 averages were similar across all the groups of mice. Thus, the CKD-induced increase in blood pressure fluctuation was attributed to the increase in the D2 averages.

The area under the curve (AUC) of D1 was larger in the daytime but smaller in the nighttime than the AUC of D2 (Supplementary Figs. S1–S12). Considering that mice are nocturnal animals and that physical activity increases blood pressure, these findings can be explained by assuming that D1 and D2 may represent blood pressure when mice are physically inactive and active, respectively. As the telemetry system monitored not only blood pressure but also physical activity (horizontal movements) of the mouse, we extracted the blood pressure data when the mouse was recorded as “walking” (locomoting horizontally) and found that the histograms showed unimodal normal distributions (Supplementary Figs. S1–S12). The averages of these unimodal normal distributions were similar to those of D2 within the same group (Fig. 3c,d). These observations indicate that D2 represents the blood pressure during physical activity, whereas D1 represents the blood pressure at rest. We conclude that the CKD does not affect the blood pressure levels at rest but enhances the pressor response during physical activity. The reason that the histograms of blood pressure when the mouse was recorded as “not walking” still showed binormal distributions (Supplementary Figs. S1–S12) may lie in the fact that vertical movements such as rearing and jumping were not recognized as physical activity in this telemetry system.

In *Fgf21*^−/−^ mice, however, not only the mean of the D1 averages but also the mean of the D2 averages were similar between control mice and CKD mice both in the nighttime and the daytime (Fig. 3a,b), indicating that FGF21 is necessary for CKD to enhance the pressor response during physical activity. To determine whether FGF21 alone can exert this effect independently of CKD, we administered the AAV8-FGF21 into wild-type normal mice by tail vein injection and raised circulating FGF21 levels to 2915 ± 401 pg/ml (mean ± SEM, *N* = 8), which was higher than those of wild-type CKD mice and comparable with those of *Fgf21*^−/−^ control mice injected with AAV8-FGF21 (Fig. 1j). When compared with mice administered with a control vector (a LacZ-expressing AAV vector, AAV8-LacZ), we observed increase in the D2 averages, but not the D1 averages, in the nighttime as observed in the wild-type CKD mice (Fig. 3a), although it did not reach statistical significance in the daytime (Fig. 3b).

### Sympathetic activation in CKD mice

**Figure 4:**
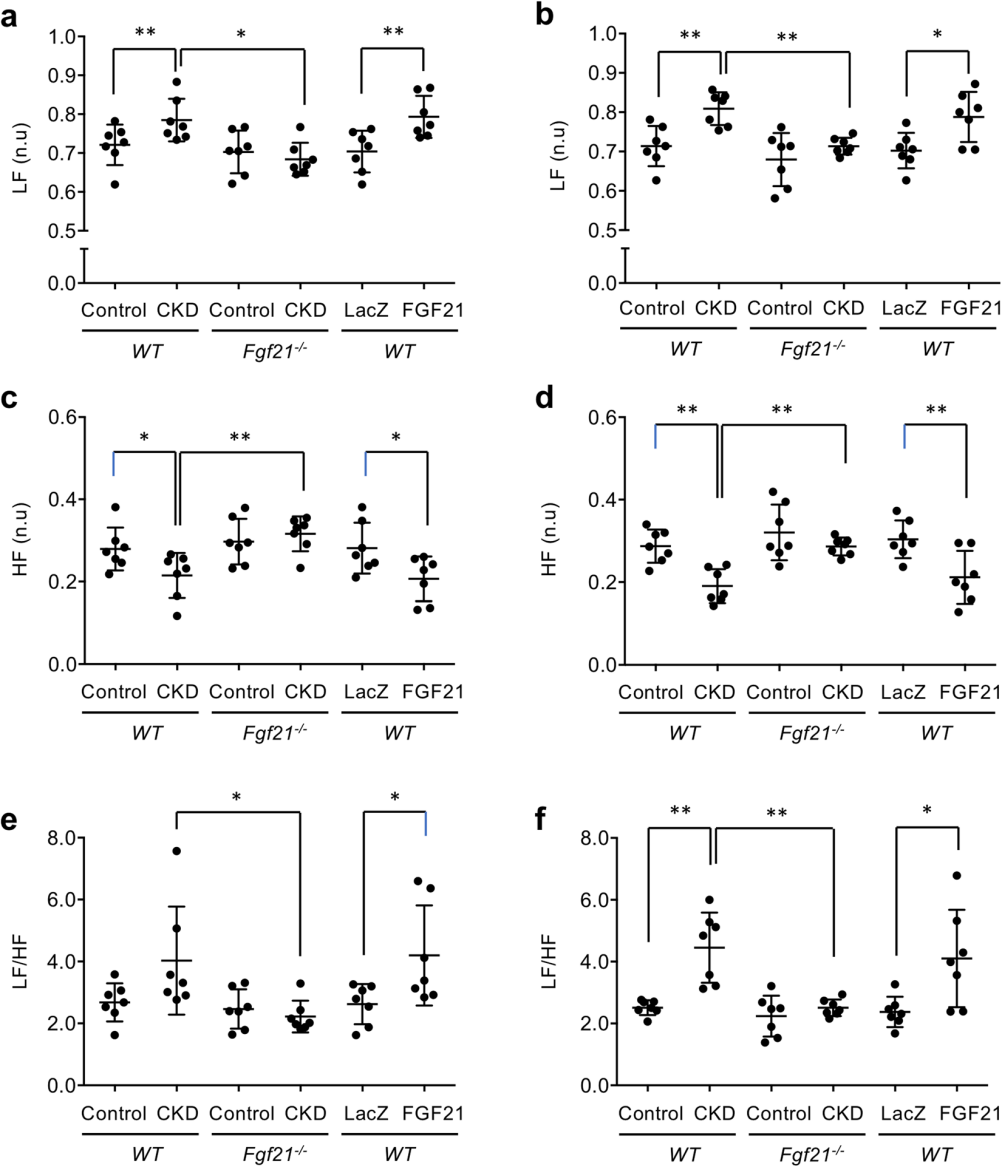
FGF21 is required to enhance sympathetic activity and suppress parasympathetic activity in CKD. The sympathetic activity (LF and LF/HF) and the parasympathetic activity (HF) during physical activity in the nighttime (**a**, **c**, **e**) and in the daytime (**b**, **d**, **f**) were calculated in wild-type control mice, wild-type CKD mice, *Fgf21*^−/−^ control mice, *Fgf21*^−/−^ CKD mice, wild-type mice injected with the AAV8-LacZ vector (LacZ) and wild-type mice injected with the AAV8-FGF21 vector (FGF21). The bars indicate mean ± SD, **P < 0.01, *P < 0.05 by t-test.

Overactivation of the sympathetic nervous system is frequently observed and contributes to augmented blood pressure responses during exercise in CKD patients^21^. Because FGF21 induces sympathetic activation^8^, we asked if increase in the sympathetic activity and reciprocal decrease in the parasympathetic activity might be augmented in CKD mice during physical activity. We extracted the electrocardiogram data when the mouse was recorded as “walking” and analyzed heart rate variability to calculate low-frequency power bandings (LF), high-frequency power bandings (HF), and the ratio of LF to HF (LF/HF). LF and LH/HF serve as indicators of the sympathetic activity, whereas HF serves as an indicator of the parasympathetic activity^22^. As expected, wild-type CKD mice had higher LF (Fig. 4a,b) and lower HF (Fig. 4c,d) than wild-type control mice, although the difference in LF/HF between the control and CKD mice did not reach statistical significance in wild-type (Fig. 4e,f). In *Fgf21*^−/−^ mice, however, introduction of CKD affected neither sympathetic nor parasympathetic activity. The LF, HF, and LF/HF levels in *Fgf21*^−/−^ CKD mice were similar to those in wild-type control mice. Again, administration of the AAV8-FGF21 to wild-type normal mice recapitulated the autonomic nerve activity similar to that observed in wild-type CKD mice (Fig. 4a–f). We conclude that FGF21 is necessary and sufficient for the augmentation of sympathetic activity and the reciprocal suppression of parasympathetic activity in this CKD model.

### Increased serum noradrenalin and corticosterone levels in CKD mice

**Figure 5:**
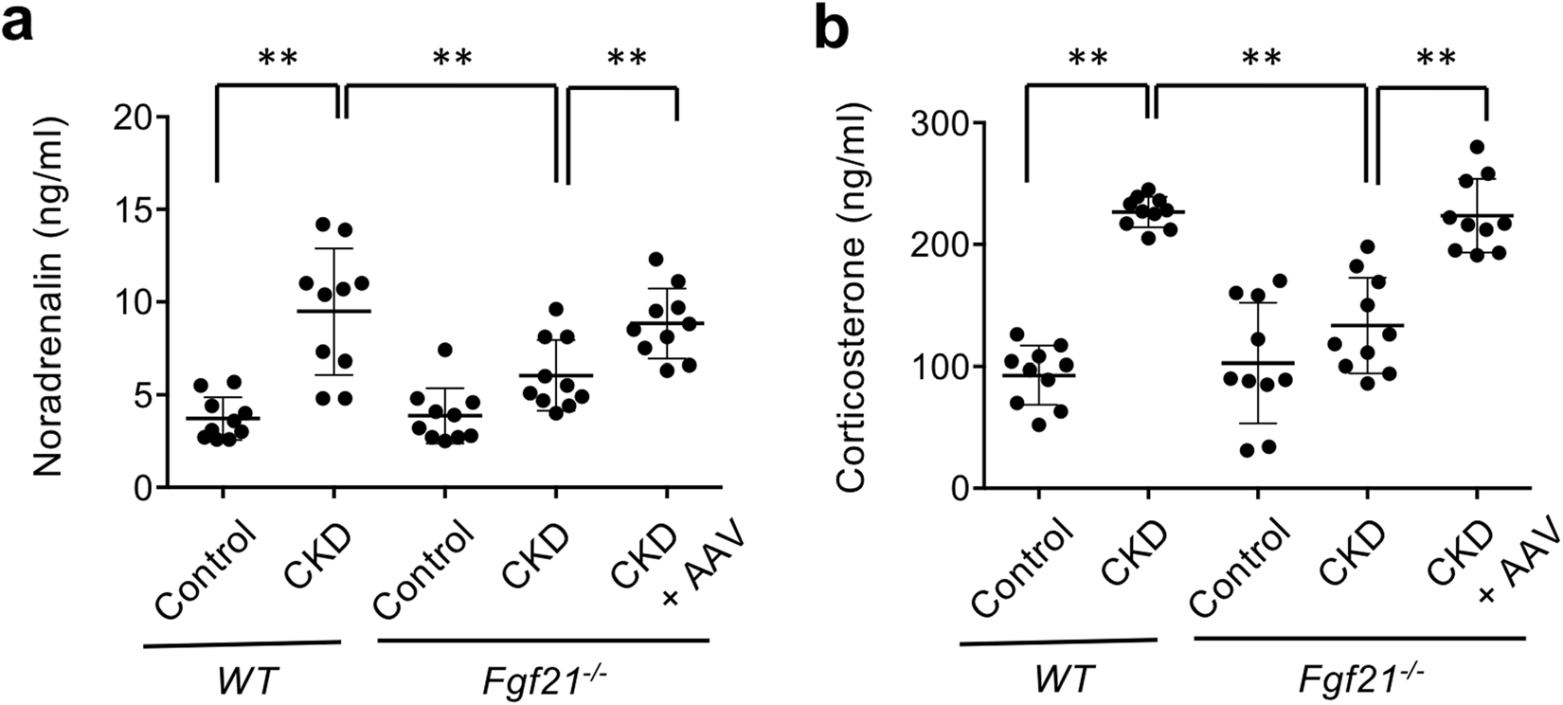
FGF21 is required to increase serum noradrenalin and corticosterone levels in CKD. Serum levels of noradrenalin (**a**) and corticosterone (**b**) were measured in wild-type control mice (WT, Control), wild-type CKD mice (WT, CKD), *Fgf21*^−/−^ control mice (*Fgf21*^−/−^, Control), *Fgf21*^−/−^ CKD mice (*Fgf21*^−/−^, CKD), *Fgf21*^−/−^ CKD mice injected with the AAV8-FGF21 vector (CKD + FGF21). The bars indicate mean ± SD, **P < 0.01 by t-test.

Consistent with the increased sympathetic activity, serum noradrenalin levels were increased in wild-type CKD mice but not in *Fgf21*^−/−^ CKD mice. When administered with the AAV8-FGF21, *Fgf21*^−/−^ CKD mice increased their serum noradrenalin to the level similar to that of wild-type CKD mice (Fig. 5a). These observations further support the conclusion that FGF21 is necessary and sufficient to increase sympathetic activity in this CKD model.

Activity of the HPA endocrine axis and serum levels of glucocorticoids are frequently increased in CKD patients^23,24^. Because FGF21 activates the HPA axis^8^, we reasoned that serum glucocorticoid levels might be increased in these CKD mice. As expected, introduction of CKD raised circulating corticosterone levels in wild-type mice but not in *Fgf21*^−/−^ mice. Again, *Fgf21*^−/−^ CKD mice administered with the AAV8-FGF21 had high serum corticosterone levels comparable with those in wild-type CKD mice (Fig. 5b), indicating that FGF21 is responsible for the increased corticosterone in this CKD model.

### Association between FGF21 and blood pressure regulation in humans

**Figure 6:**
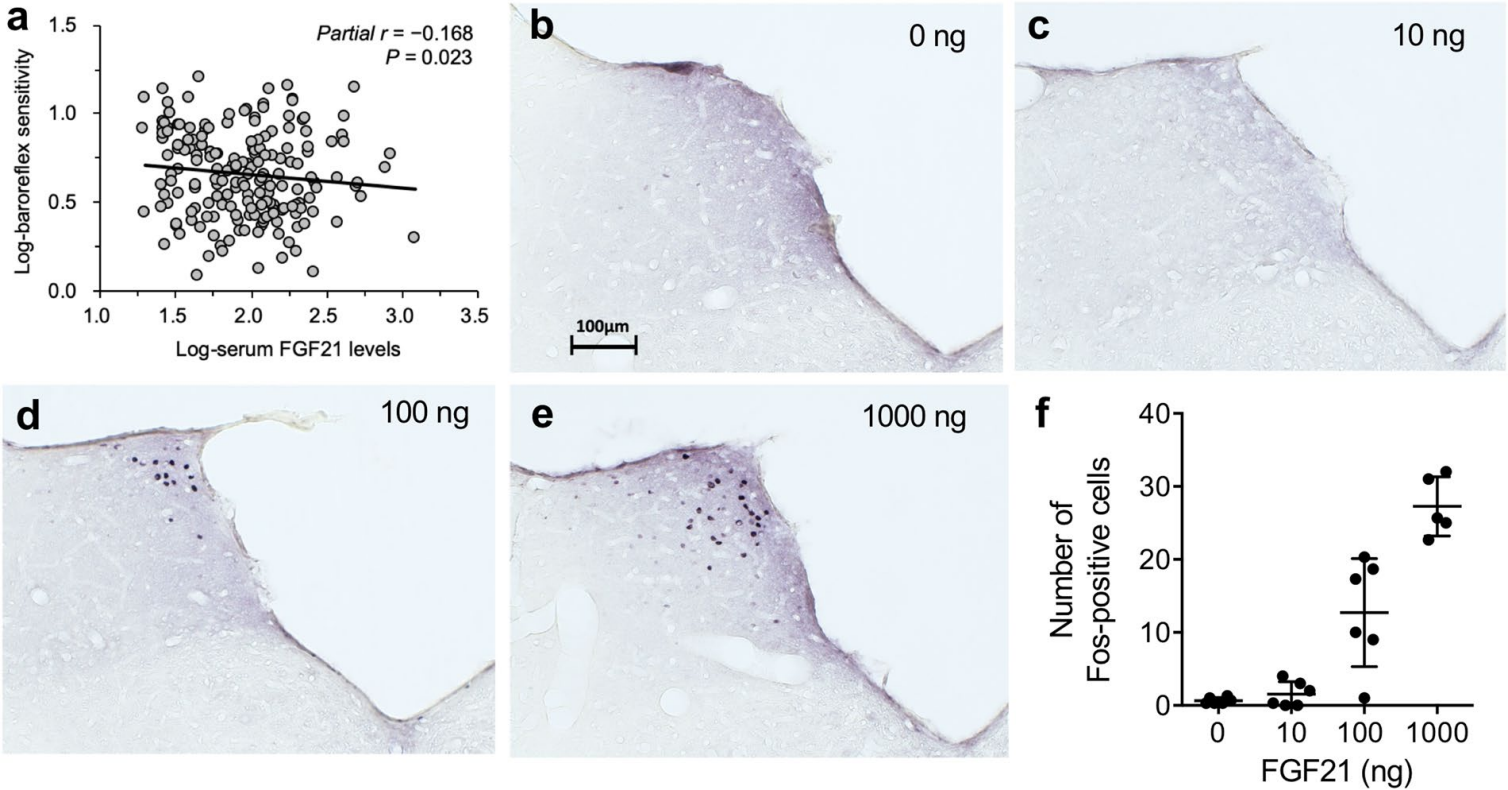
FGF21 activates NTS neurons and may suppress baroreflex. (**a**) Correlation between serum FGF21 levels and baroreflex sensitivity by Pearson correlation coefficient in 184 CKD patients. Adjusted for age and sex. Immunohistochemistry of the NTS region after intracerebroventricular injection of 0 ng (**b**), 10 ng (**c**), 100 ng (**d**), and 1000 ng (**e**) of FGF21. Bar = 100 mm. (**f**) The number of c-Fos positive cells was increased in an FGF21 dose-dependent manner. *P* < 0.0001 by one-way ANOVA.

Baroreflex buffers sympathetic activation and blood pressure elevation during physical activity^25^. Because CKD patients have impaired baroreflex sensitivity (BRS)^26^, the fact that FGF21 is primarily responsible for augmented sympathetic activation and pressor response during physical activity raised the possibility that circulating FGF21 levels might negatively correlate with BRS in CKD patients. To test this possibility, we performed an observational study by enrolling 185 non-dialysis CKD patients to measure serum FGF21 and BRS together with several other clinical parameters (Supplementary Table S7). As expected, BRS was correlated negatively with serum FGF21 levels (Fig. 6a). Multiple linear regression analysis identified serum FGF21 levels as one of the independent determinants of BRS (Table 1).

**Table 1:** Independent determinates of baroreflex sensitivity.

Variables	B	±	SE	β	*P*
**R**^**2**^** = 0.124**					
Sex (woman)	−0.154	±	0.035	−0.310	<0.001
Age (years)	−0.005	±	0.002	−0.168	0.017
Serum FGF21 levels (pg/mL)*	−0.110	±	0.048	−0.161	0.023

Covariates in the stepwise linear regression model included age, gender (woman), body mass index, visceral fat (*N* = 184), eGFR (average of estimated glomerular filtration rate calculated from serum creatinine and that from cystatin C), current smoking status, anti-hypertensive medication, and serum FGF21 levels. *Log-transformed.

## Discussion

Because FGF21 functions as a longevity hormone that extends life span when overexpressed in mice^17^, the fact that CKD mice and CKD patients have high serum FGF21 levels may appear inconsistent with their high mortality. This apparent paradox can be explained by the fact that FGF21 induces stress responses required to survive CKD. Namely, the higher circulating FGF21 levels can be viewed as a consequence of the severer life-threatening “stress” and thus associated with the poorer prognosis. This notion is supported by a clinical study showing that the dialysis patients with serum FGF21 levels higher than the median had higher mortality than the others^14^.

It remains to be determined how hepatocytes sense the “stress” caused by CKD and induce FGF21 expression/secretion. In rats, dietary phosphate load was reported to induce expression of the PPARα target genes including the *Fgf21* gene^27^, and thus may contribute to the high serum FGF21 levels in the CKD mice in this study. However, this discussion may not be applicable to CKD patients, because CKD patients often restrict their phosphate intake as part of diet therapy but still have high serum FGF21 levels. The fact that CKD patients often exhibit dyslipidemia and have high serum levels of free fatty acids^28^ may contribute to the high serum FGF21 levels, because free fatty acids are endogenous ligands for PPARα^29^.

The cause of death of *Fgf21*^−/−^ CKD mice also remains to be specified. As mentioned earlier, their cause of death is likely an unknown extrarenal complication(s) that FGF21 would have counteracted, at least in part. Some *Fgf21*^−/−^ CKD mice gradually lost body weight and became inactive before they reached an endpoint. The other *Fgf21*^−/−^ CKD mice were indistinguishable from wild-type CKD mice until censored at 18 weeks of age in this study. Further studies are needed to clarify the cause of this phenotypic variability.

Bookout *et al*. demonstrated that transgenic mice that overexpress FGF21 exhibited disturbed circadian rhythm as determined by spontaneous wheel-running behavior^8^. On the other hand, mice with high serum FGF21 levels caused by either CKD or FGF21 overexpression exhibited normal circadian rhythm as determined by diurnal blood pressure fluctuation (Fig. 2). Because circadian rhythm is regulated not only by FGF21 but also by many other factors, effects of FGF21 on different outputs of circadian rhythm can be variable. It is possible that the circadian rhythm of the wheel-running behavior may be affected primarily by activation of SCN with FGF21, whereas circadian blood pressure fluctuation may be affected by additional factors, such as diurnal changes of the renin-angiotensin system^30^, which may predominate over the effect of FGF21.

Baroreflex is composed of stretch-sensitive baroreceptors in blood vessels and afferent nerves projecting from baroreceptors to neurons in the nucleus of solitary tract (NTS) in the brainstem, which serves as the center for the regulation of both sympathetic and parasympathetic activity to buffer blood pressure fluctuation^31^. Because βKlotho, the obligate co-receptor for FGF21, is expressed in the NTS^8^, it is intriguing to speculate that FGF21 might directly act on the NTS neurons to attenuate baroreflex. In fact, administration of recombinant FGF21 protein to rats by intracerebroventricular injection induced c-Fos expression in the neurons in the rostral NTS region (Fig. 6b–f). Although it is beyond the scope of this study to elucidate the effect of FGF21 on the NTS and baroreflex, these findings have raised the possibility that increased FGF21 in CKD might suppress baroreflex through directly acting on the NTS neurons, thereby enhancing sympathetic activity and pressor response during physical activity. It is also possible to argue that the cause of impaired baroreflex in CKD may lie not only in the center but also in the periphery. Vascular stiffness associated with CKD progression likely restrains changes in dimensions of the blood vessel wall and impairs the function of stretch-sensitive baroreceptors. In fact, when the carotid-femoral pulse wave velocity (PWV), a clinical parameter for vascular stiffness, was forcibly entered in the multiple linear regression analysis in our observational study, PWV was selected as an independent determinant of the BRS (Supplementary Table S8). In our mouse study, however, wild-type CKD mice showed greater pressor response and sympathetic activity during physical activity than *Fgf21*^−/−^ CKD mice despite the fact that their vascular stiffness appeared comparable, because expression levels of markers for vascular calcification were not different between the wild-type CKD mice and the *Fgf21*^−/−^ CKD mice. Furthermore, the pressor response during physical activity and sympathetic activity of *Fgf21*^−/−^ CKD mice was significantly enhanced when they were treated with AAV8-FGF21 without any changes in the expression levels of markers for vascular calcification (Fig. 1g–i). These observations can be explained by the hypothesis that FGF21 may directly act on the NTS neurons and suppress baroreflex. Further studies are necessary to validate this hypothesis.

In conclusion, the present study has provided new insights into the role that FGF21 plays in CKD pathophysiology. Specifically, we showed that FGF21 conferred survival benefit on CKD mice (Fig. 1k). We also showed that FGF21 induced several CKD complications including sustained sympathetic hyperactivity and augmented pressor response during physical activity (Figs. 3–5). Although these complications induced by FGF21 are regarded as the cardiovascular risk^32^, CKD mice would have died earlier if FGF21 had not been increased. We construe these findings that CKD mice increase FGF21 to acquire the survival benefit in exchange for these complications. Therefore, suppression of the sympathetic tone in CKD patients may reduce the cardiovascular risk, especially when serum FGF21 levels are elevated. In fact, administration of beta-blockers was shown to improve all-cause mortality in patients with stages 3 to 5 CKD^33^. The present study may encourage clinical studies to determine whether CKD patients with high serum FGF21 levels may derive more benefits from beta-blockers than those with low FGF21.

## Methods

All animal experiments were approved by the institutional animal care and use committee (IACUC) from Jichi Medical University, and carried out in accordance with the “Fundamental guidelines for proper conduct of animal experiment and related activities in academic research institutions under the jurisdiction of the Ministry of Education, Culture, Sports, Science and Technology, Japan”. All clinical studies were conducted in accordance with the Declaration of Helsinki. The study protocols were approved by Ethical Committee of the University of Tsukuba and Jichi Medical University. All participants provided a written informed consent to participate in this study.

### A mouse CKD model

Wild-type mice (C57BL/6 J males) and *Fgf21*^−/−^ mice (C57BL/6 J congenic males) were subjected to right uninephrectomy at 8 weeks of age. After 4 weeks of the recovery period, they were placed on a high phosphate diet containing 2.0% inorganic phosphate at 12 weeks of age. Within 5 weeks after the dietary phosphate load, these mice develop renal tubular damage and interstitial inflammation and fibrosis as shown in Fig. 1. These mice were designated as CKD mice. As a control for these CKD mice, age- and sex-matched wild-type mice and *Fgf21*^−/−^ mice were subjected to sham operation (laparotomy alone) at 8 weeks of age and then placed on a regular diet containing 0.35% inorganic phosphate at 12 weeks of age. All mice were then transferred individually to metabolic cages to measure food and water consumption and to collect urine for 3 days and then sacrificed to harvest blood and tissues including kidney and aorta. All samples were snap-frozen in liquid nitrogen and then stored at −80 °C until used for the analyses.

### AAV vectors

The AAV8 vectors expressing mouse FGF21 or LacZ under the control of the CAG promotor (chicken β-actin promoter with the CMV immediate-early enhancer) were prepared according to the method previously described^34^. Briefly, 293 cells were transfected simultaneously with adenovirus helper plasmid, AAV type 2 helper plasmid, and the LacZ or FGF21 plasmid by calcium phosphate transfection. Three days later, these 293 cells were subjected to three freeze-thaw cycles to harvest the AAV vectors. After purification by cesium chloride density gradient centrifugation, the titers of the AAV vectors were measured by RT-qPCR. Each mouse was injected with 1.0 × 10^9^ viral genomes (vg) of AAV8-FGF21 or AAV8-LacZ.

### Kaplan-Meier analysis

Twenty wild-type CKD mice and 40 *Fgf21*^−/−^ CKD mice were prepared as described above. At 12 weeks of age, 20 *Fgf21*^−/−^ CKD mice were injected with 1.0 × 10^9^ vg of AAV8-FGF21. The other 20 *Fgf21*^−/−^ CKD mice were injected with 1.0 × 10^9^ vg of AAV8-LacZ. All the mice were inspected every day to identify moribund mice that fulfilled the criteria for euthanasia approved by the IACUC. Such mice were recorded dead on the day of euthanasia. The other mice that reached 18-week-old were censored.

### Quantitative RT-PCR

Frozen mouse tissues were homogenized with RNAiso Plus (TaKaRa). The lysate was transferred to a microcentrifuge tube and extracted with chloroform. RNA in the aqueous phase was precipitated with isopropanol, washed with 75% ethanol, and dissolved in RNase-free water. Reverse transcription of RNA (0.4 μg) was carried out using ReverTra Ace qPCR RT Master Mix with gDNA Remover (Toyobo, FSQ-301) according to the manufacturer’s protocol. Quantitative RT-PCR reactions contained 20 ng of cDNA, 410 nM of each primer, and 6 μl of SYBR Green PCR Master mix (THUNDERBIRD SYBR qPCR Mix QPS-201, Toyobo) in a total volume of 12 μl. The PCR reaction (95 °C for 1 minute followed by 45 cycles of 95 °C for 10 seconds, 60 °C for 40 seconds) was performed on Roche LC480 system. Relative mRNA levels were calculated by the comparative threshold cycle method using cyclophilin as an internal control. The sequence of the primers for osteopontin, Ngal, MCP1, Runx2, and osteocalcin were shown in Supplementary Table 3.

### Blood and urine analyses

All mice were starved for 6 hours (9:00AM~3:00PM) before blood sampling at 3:00PM. Plasma FGF21 levels were measured using FGF21 Mouse/Rat ELISA Kit (Funakoshi BLM RD291108200R) according to the manufacturer’s protocol. Serum and urine phosphate levels were measured using Fuji Dri-Chem slides and the analyzer (Dri-Chem NX500V, Fuji). Serum and urine creatinine levels were measured using Determiner L CRE (Kyowa Medex, Tokyo, Japan). Plasma corticosterone and noradrenalin levels were measured using Corticosterone ELISA Kit (ab108821 Abcam plc, Cambridge, UK) and Norepinephrine ELISA Kit (Abnova KA1891), respectively.

### Telemetry analysis

Arterial pressure and electrocardiogram (ECG) were measured continuously over 48 hours using a mouse telemetry system (DSI PhysioTel HD-X11 transmitter and Ponemah v5.20 software, Data Sciences International, St. Paul, MN) under the unanesthetized and unrestraint condition. The transmitters were transplanted to mice that weighed over 21 g at 16 weeks of age. The pressure catheter was inserted at the right carotid artery and advanced to the aortic arch for measurement of arterial pressure. The biopotential probes were placed subcutaneously at the right pectoral muscle and at the left caudal rib region for ECG recording. The body of the transmitter was implanted subcutaneously at the left flank. These mice were allowed to recover from the surgery for a week and then transferred individually to cages on the telemetry receivers to record systolic, diastolic, and average blood pressure and heart rate variability. All the mice were housed under 12/12 hour light/dark cycle with lights on at 7:00AM and given ad libitum access to food and water.

### Blood pressure analysis

Data segments of 30 sec were used for histograms with 1 mmHg bins. The data segments associated with activity were used for ‘walking” histograms. The data segments associated with no activity were used for ‘not walking” histograms (Supplementary Fig. S1–S12). The curve fitting for sum of two normal distributions and calculation of the average and standard deviation of each normal distribution were done in Graphpad Prism 6 or manually by the least squares method.

### Heart rate variability

HR variability were evaluated using spontaneous changes in HR as previously reported^35^, except that 1) beat-to-beat values of detected R-R intervals were resampled at 10 Hz, 2) data segments of 30 sec were used for spectral analysis, 3) the power in the frequency range 0.4–1.5 Hz was calculated as low frequencies (LF).

### Observational study

Total of 218 adults (≥45 years old) who fulfilled at least one of the following two criteria were included in this study: (1) estimated glomerular filtration rate (eGFR) < 90 mL/min/1.73 m^2^. (2) micro- or macroalbuminuria ≥30 mg/g creatinine. After excluding participants who had missing values of required measurements (n = 29) or who had breakfast or smoking in the morning on the day of measurements (n = 4), we enrolled 184 adults (Supplementary Table 1). The BRS was defined as the transfer function gain calculated from the cross-spectral between systolic blood pressure (SBP) and R-R interval in the low frequency range^36^. Briefly, continuous beat-to-beat SBP and R-R interval were recorded for 5 minutes in the spine position. Spectral powers of SBP and R-R interval were estimated from low-frequency range (0.05–0.15 Hz)^36,37^. Height, body mass, and waist circumference were evaluated with the participants barefoot, wearing only light clothing. Visceral fat was assessed by using the dual-impedance analysis method (HSD-2000; Omron Healthcare, Kyoto, Japan). Brachial systolic blood pressure, diastolic blood pressure, and carotid-femoral pulse wave velocity (PWV) were simultaneously measured using semi-automatic vascular testing device with electrocardiogram and oscillometric extremities cuffs (form PWV/ABI, Colin Medical technology, Komaki, Japan) as previously described^38^. eGFR was calculated by the Japanese eGFR equations based on standardized serum creatinine or cystatin C as follows: eGFR_cr_ (mL/min/1.73 m^2^) = 194 × serum creatinine^−1.094^ × Age^−0.287^ × 0.739 (if female), eGFR_cys_ (mL/min/1.73 m^2^) = [104 × serum cystatin C^−1.019^ × 0.996 Age × 0.929 (if female)] − 8. To improve estimated accuracy, the average values of eGFRcr and eGFRcys were used in this study. The serum FGF21 levels were assessed by using a sandwich enzyme-linked immunosorbent assay (ELISA) kit (Bio Vendor; Modrice, Czech Republic)^14^. All analyses were conducted using SPSS Statistics Version 25.0 (IBM, Tokyo, Japan). Data were expressed as mean ± SD or numbers with percentages. Variables with a skewed distribution were log-transformed and standardized to a normal distribution. Independent determinants of the BRS were examined using multiple regression analyses with the stepwise procedure. In the initial stepwise model (Model 1, Table 1), the potential covariates and serum FGF21 levels were entered. In the subsequent model (Model 2, Supplementary Table S2), the variables that independently associated with the BRS in the Model 1 were forcibly entered together with the carotid-femoral PWV. Statistical significance was defined as a P value less than 0.05.

### Surgery and intracerebroventricular (i.c.v.) injection

Surgery and i.c.v. injection of FGF21 was performed as previously described^39^. Briefly, male rats (9 weeks old, slc:Wistar; SLC Japan, Shizuoka, Japan) were anesthetized with Avertin (200 mg/ kg BW, i.p.; tribromoethanol; WAKO Pure Chemical Industries Ltd, Osaka, Japan) and placed in a stereotaxic frame. Stainless steel guide cannulae (23-gauge) were inserted into the right lateral cerebral ventricle (0.6 mm caudal to the bregma, 1.6 mm lateral to the midline and 4.5 mm below the skull) and secured to the skull with screws and dental cement. Rats were allowed to recover for 2 weeks. Rats were injected i.c.v. with FGF21 (0, 10, 100, or 1,000 ng/5 μL, 5–6 rats per dose) via inner cannulae (30-gauge).

### Immunohistochemistry

Rat brains were harvested 100 minutes after FGF21 injection and fixed with 4% paraformaldehyde followed by sucrose cryopreservation. The rostral NTS were cut coronally at 30 μm with a cryomicrotome at 120 μm intervals and subjected to immunohistochemical detection for c-Fos protein as previously described^40^.

## Supplementary material

Supplementary information
