# Supplementary material for: Increased fibroblast growth factor-21 in chronic kidney disease is a trade-off between survival benefit and blood pressure dysregulation
Source: Sci Rep. 2019 Dec 17;9:19247. doi: 10.1038/s41598-019-55643-4 (PMC6917750; doi:10.1038/s41598-019-55643-4)
Supplement: Supplementary file 1 — Supplementary information [file 41598_2019_55643_MOESM1_ESM.pdf]

Supplementary information

Increased fibroblast growth factor-21 in chronic kidney disease is a trade-off between survival benefit and blood pressure dysregulation

Toshihiro Nakano[1,2], Kazuhiro Shiizaki[1], Yutaka Mirua[1], Masahiro Matsui[3], Keisei Kosaki[4,5,6], Shoya Mori[3], Kunihiro Yamagata[7], Seiji Maeda[5], Takuya Kishi[8], Naoki Usui[9], Masahide Yoshida[9], Tatsushi Onaka[9], Hiroaki Mizukami[10], Ruri Kaneda[1], Kazunori Karasawa[2], Kosaku Nitta[2], Hiroshi Kurosu[1], and Makoto Kuro-o[1,11,12,*]

[1]Division of Anti-aging Medicine, Center for Molecular Medicine, Jichi Medical University, Tochigi, Japan.

[2]Department of Medicine IV, Tokyo Women's Medical University, Tokyo, Japan.

[3] Graduate School of Comprehensive Human Sciences, University of Tsukuba, Ibaraki, Japan

[4] Faculty of Sport Sciences, Waseda University, Saitama, Japan

[5] Faculty of Health and Sport Sciences, University of Tsukuba, Ibaraki, Japan

[6] Japan Society for the Promotion of Science, Tokyo, Japan

[7] Department of Nephrology, Faculty of Medicine, University of Tsukuba, Ibaraki, Japan

[8] Faculty of Health and Welfare Sciences in Fukuoka, International University of Health and Welfare, Fukuoka, Japan.

[9] Division of Brain and Neurophysiology, Department of Physiology, Jichi Medical University, Tochigi, Japan.

[10] Division of Genetic Therapeutics, Center for Molecular Medicine, Jichi Medical University, Tochigi, Japan.

[11] Charles and Jane Pak Center for Mineral Metabolism and Clinical Research, Department of Internal Medicine, University of Texas Southwestern Medical Center, Dallas, Texas, USA.

[12] AMED-CREST, Japan Agency for Medical Research and Development, Tokyo, Japan.

*Correspondence: Makoto Kuro-o, Division of Anti-aging Medicine, Center for Molecular Medicine, Jichi Medical University, 3311-1 Yakushiji, Shimotsuke, Tochigi 329-0498, Japan. Email: mkuroo@jichi.ac.jp

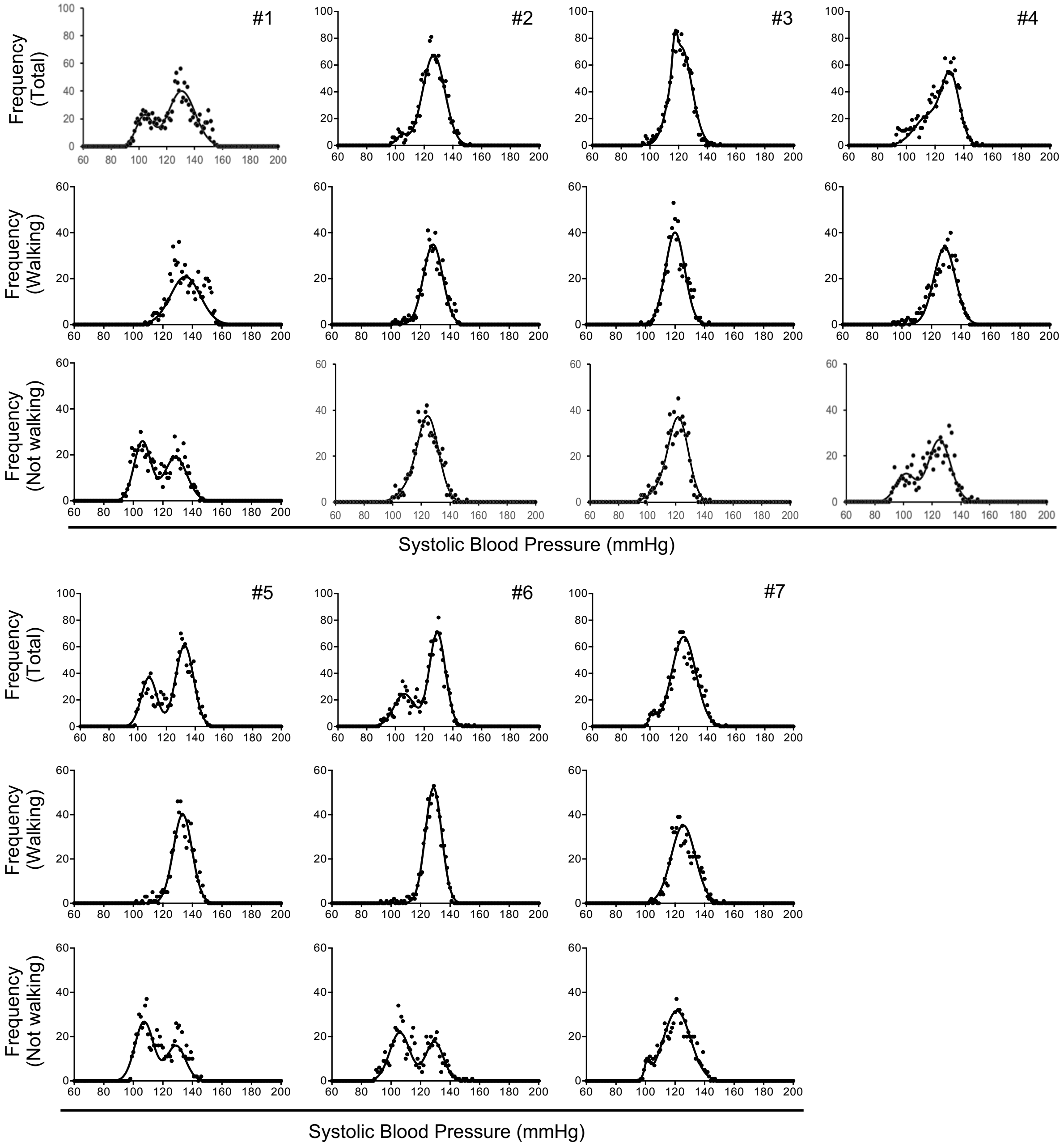

Supplementary Figure S1. Histograms of systolic blood pressure (SBP) in wild-type control mice during nighttime. SBP (top panels), SBP when the mouse was walking (moving horizontally, middle panels), and SBP when the mouse was not walking (moving vertically or not moving, bottom panels) were shown ($N = 7$). The mean and the standard deviation of individual mice were shown in Supplementary Table S1-S3.

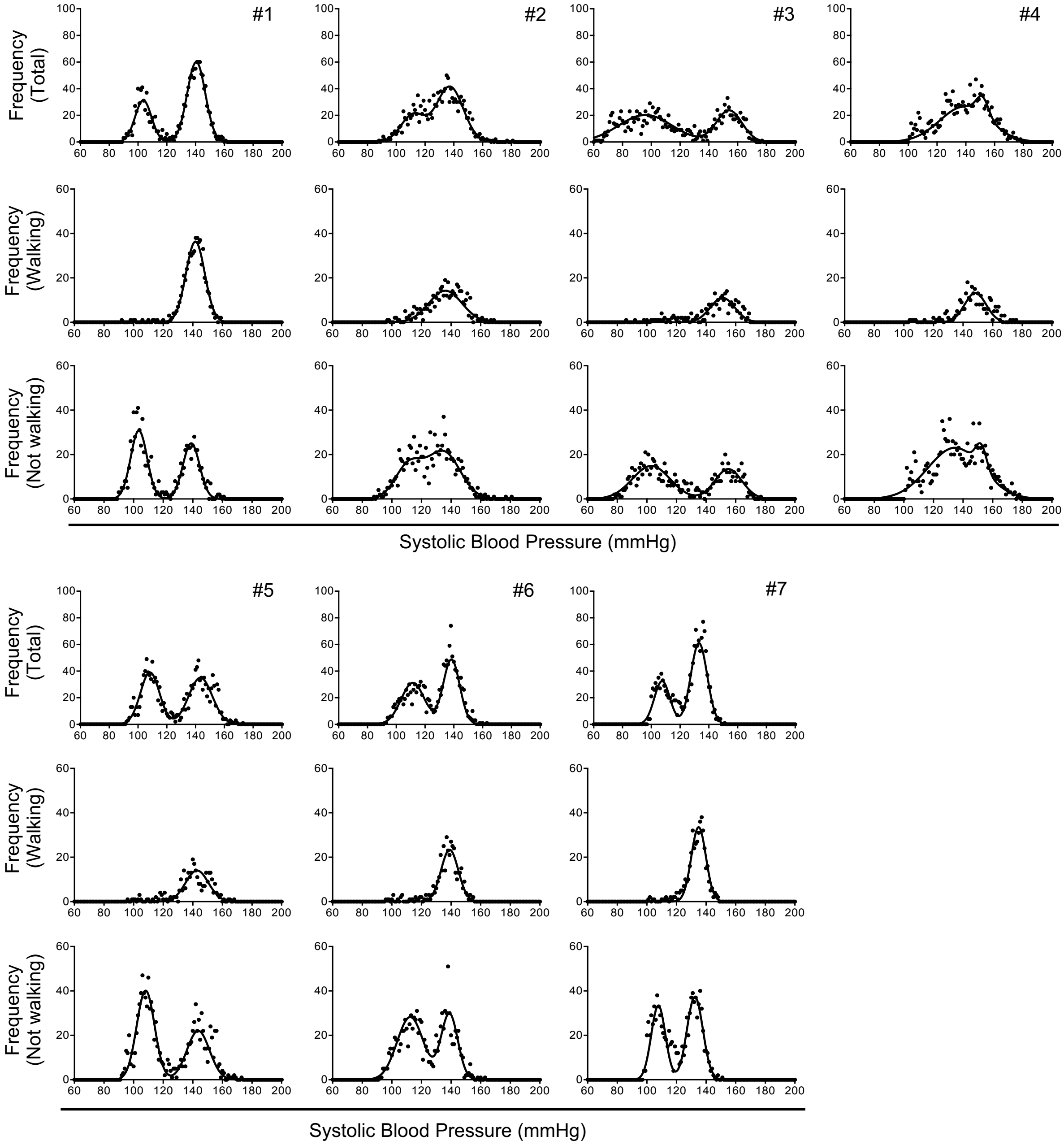

Supplementary Figure S2. Histograms of systolic blood pressure (SBP) in wild-type CKD mice during nighttime. SBP (top panels), SBP when the mouse was walking (moving horizontally, middle panels), and SBP when the mouse was not walking (moving vertically or not moving, bottom panels) were shown (N = 7). The mean and the standard deviation of individual mice were shown in Supplementary Table S1-S3.

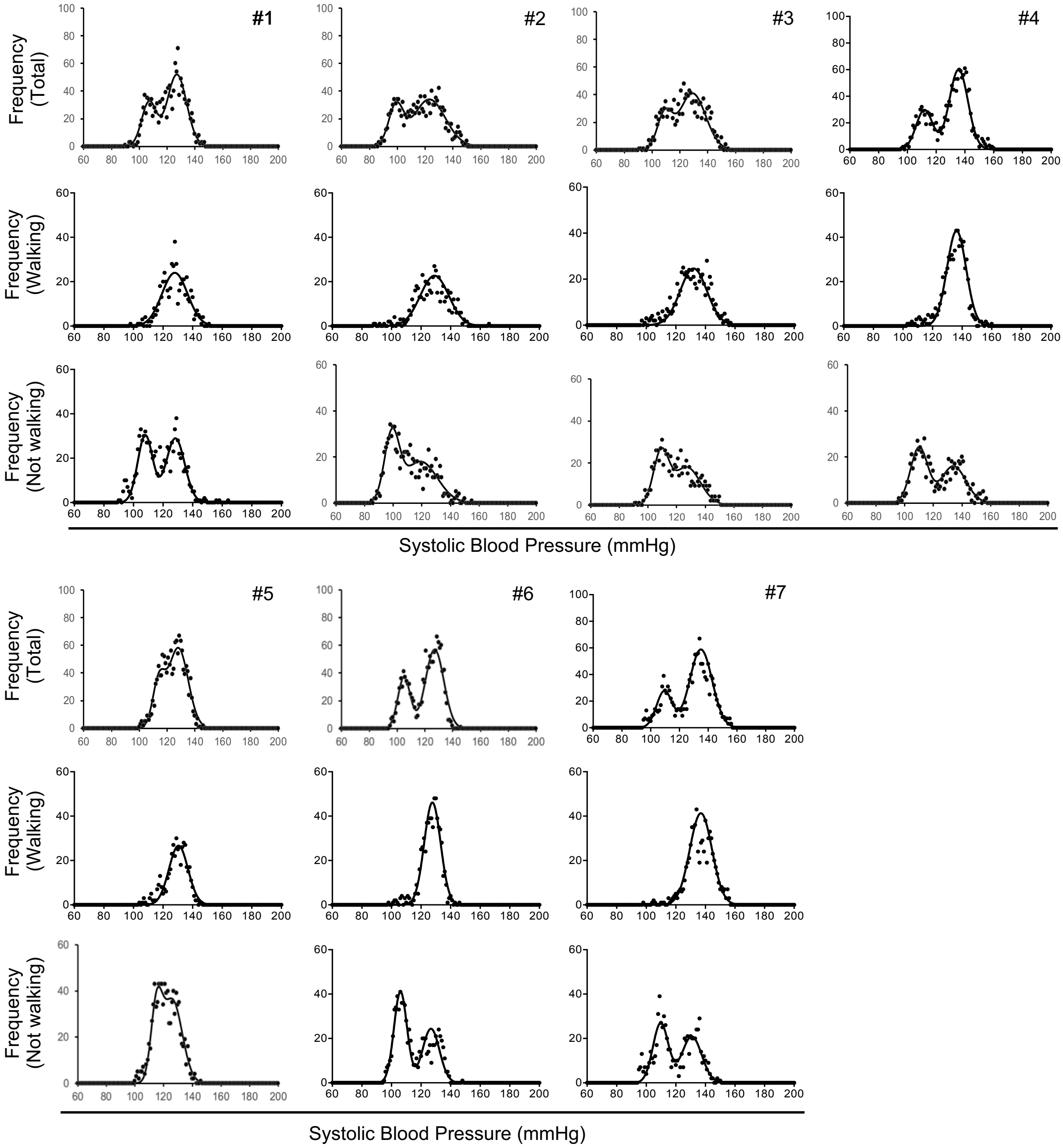

Supplementary Figure S3. Histograms of systolic blood pressure (SBP) in *Fgf21*$^{-/-}$ control mice during nighttime. SBP (top panels), SBP when the mouse was walking (moving horizontally, middle panels), and SBP when the mouse was not walking (moving vertically or not moving, bottom panels) were shown ($N = 7$). The mean and the standard deviation of individual mice were shown in Supplementary Table S1-S3.

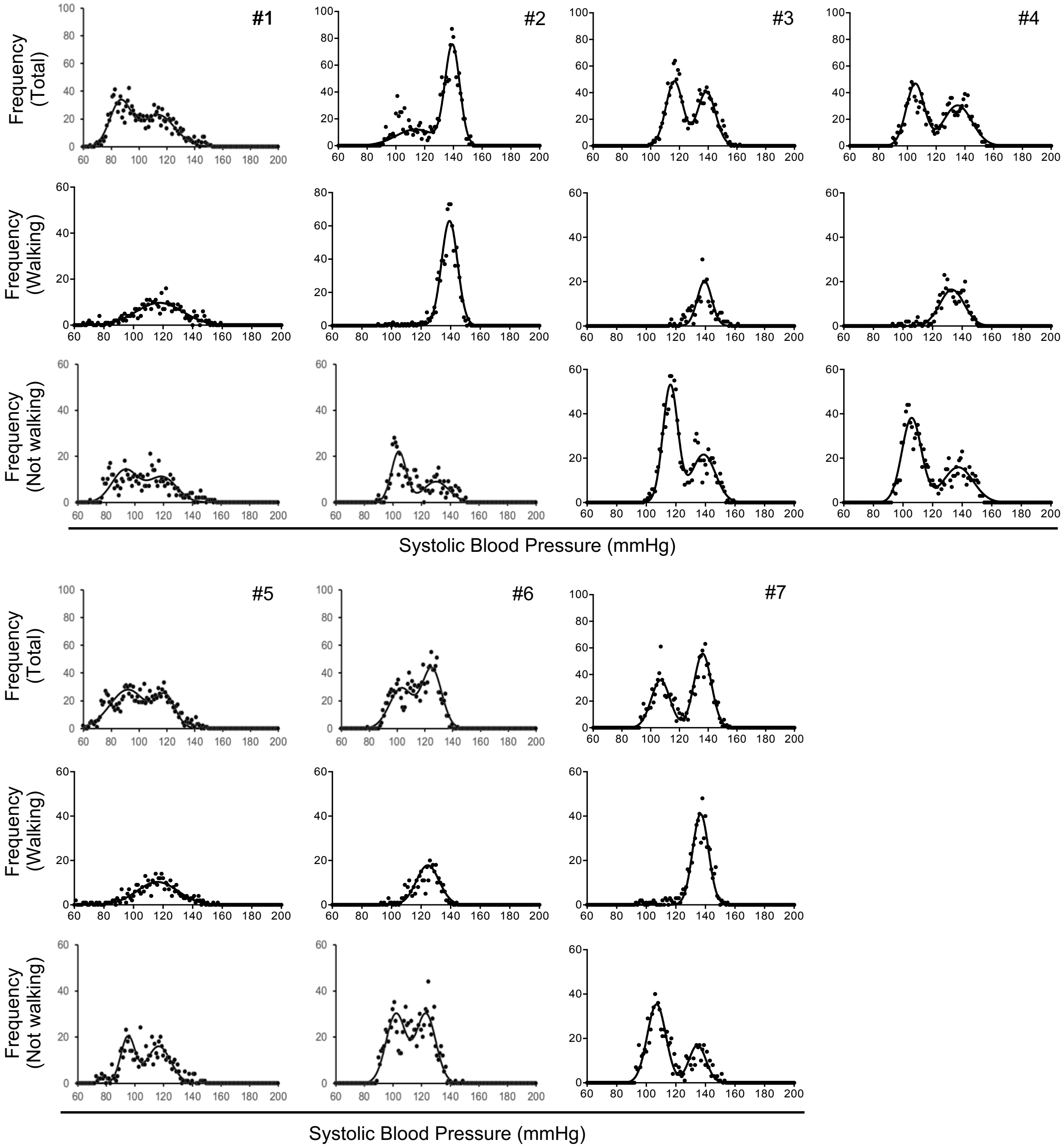

Supplementary Figure S4. Histograms of systolic blood pressure (SBP) in *Fgf21*$^{-/-}$ CKD mice during nighttime. SBP (top panels), SBP when the mouse was walking (moving horizontally, middle panels), and SBP when the mouse was not walking (moving vertically or not moving, bottom panels) were shown (N = 7). The mean and the standard deviation of individual mice were shown in Supplementary Table S1-S3.

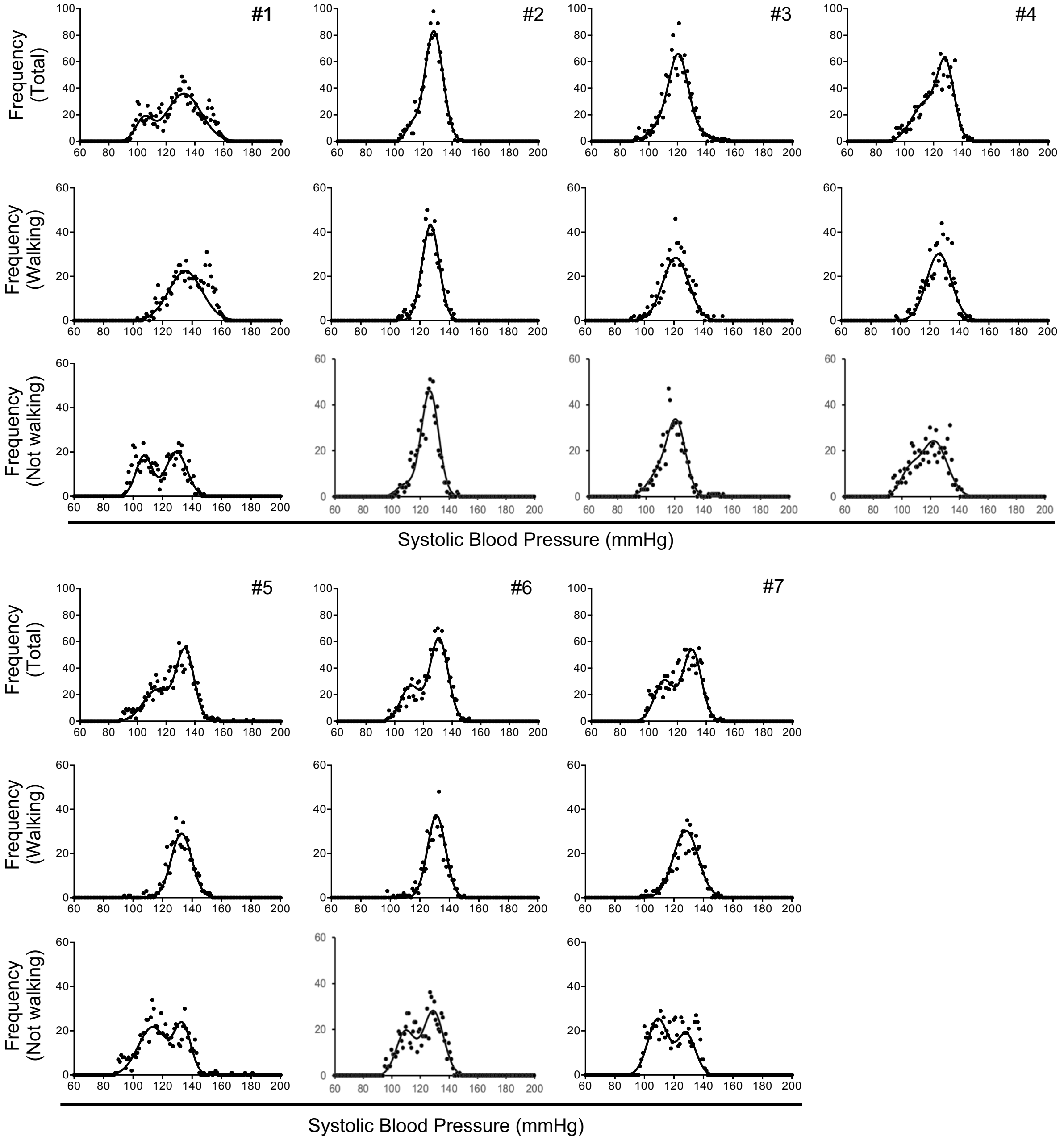

Supplementary Figure S5. Histograms of systolic blood pressure (SBP) in wild-type mice injected with the AAV8-LacZ during nighttime. SBP (top panels), SBP when the mouse was walking (moving horizontally, middle panels), and SBP when the mouse was not walking (moving vertically or not moving, bottom panels) were shown (N = 7). The mean and the standard deviation of individual mice were shown in Supplementary Table S1-S3.

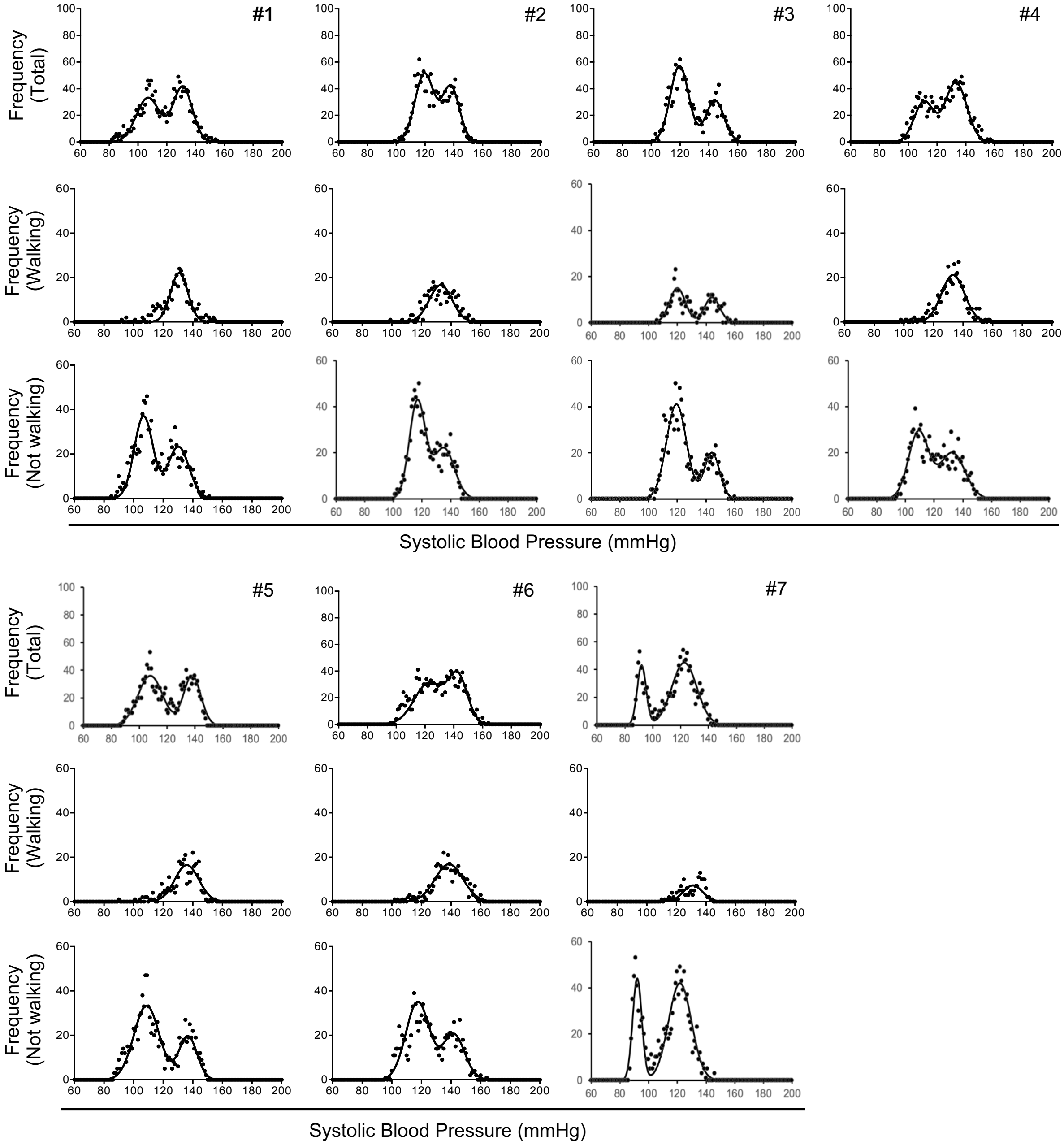

Supplementary Figure S6. Histograms of systolic blood pressure (SBP) in wild-type mice injected with the AAV8-FGF21 during nighttime. SBP (top panels), SBP when the mouse was walking (moving horizontally, middle panels), and SBP when the mouse was not walking (moving vertically or not moving, bottom panels) were shown (N = 7). The mean and the standard deviation of individual mice were shown in Supplementary Table S1-S3.

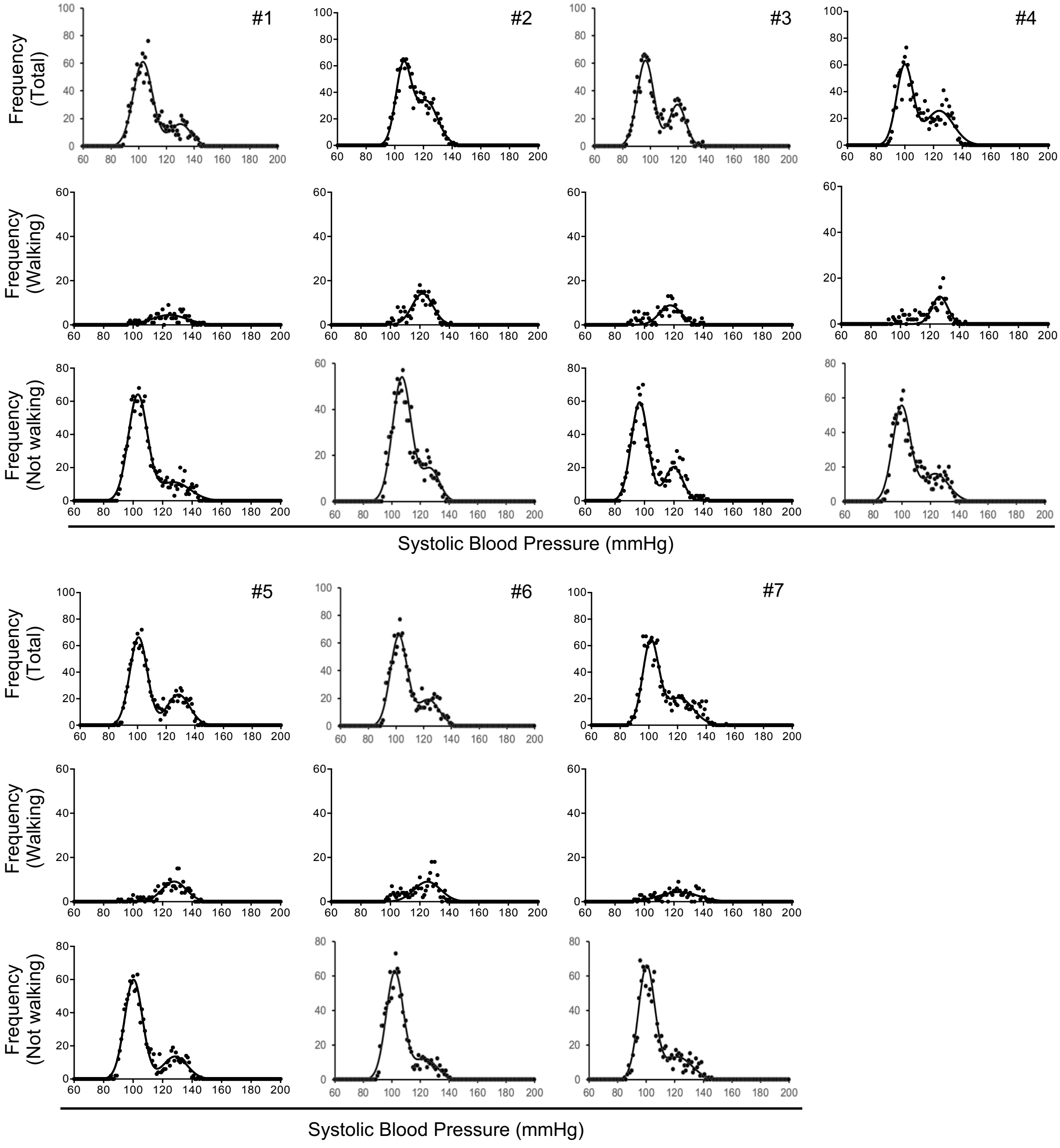

Supplementary Figure S7. Histograms of systolic blood pressure (SBP) in wild-type control mice during daytime. SBP (top panels), SBP when the mouse was walking (moving horizontally, middle panels), and SBP when the mouse was not walking (moving vertically or not moving, bottom panels) were shown ($N = 7$). The mean and the standard deviation of individual mice were shown in Supplementary Table S4-S6.

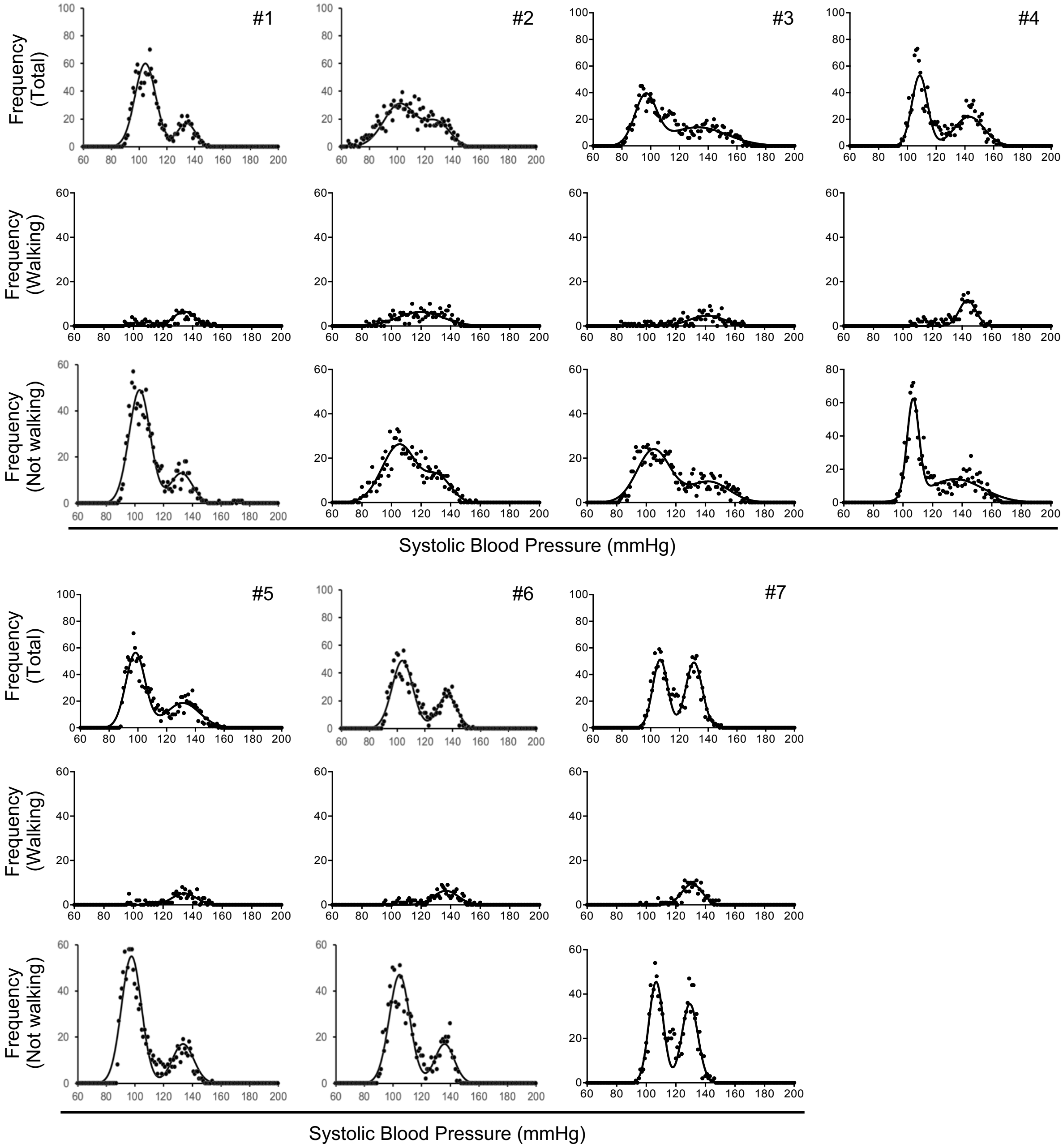

Supplementary Figure S8. Histograms of systolic blood pressure (SBP) in wild-type CKD mice during daytime. SBP (top panels), SBP when the mouse was walking (moving horizontally, middle panels), and SBP when the mouse was not walking (moving vertically or not moving, bottom panels) were shown (N = 7). The mean and the standard deviation of individual mice were shown in Supplementary Table S4-S6.

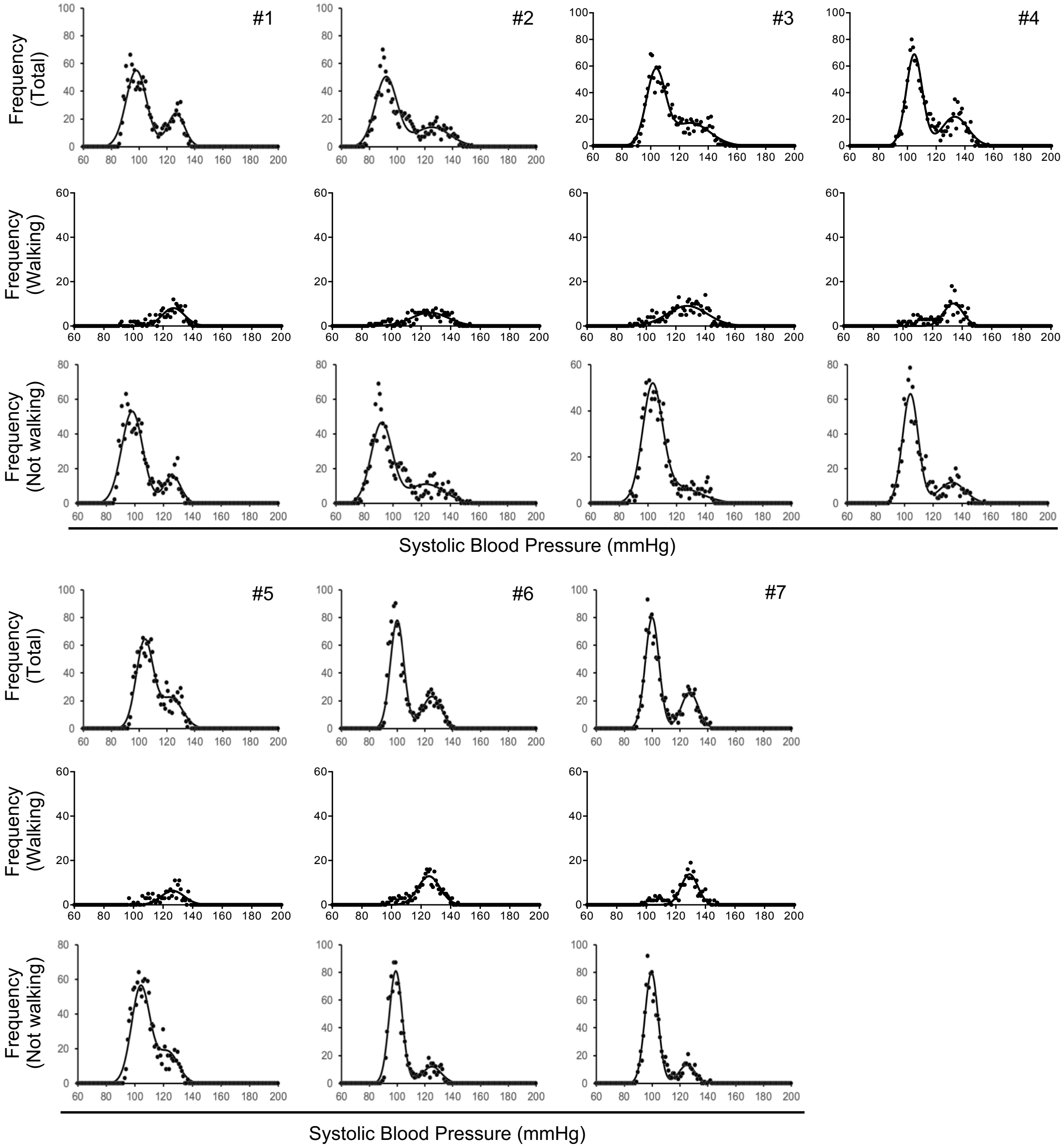

Supplementary Figure S9. Histograms of systolic blood pressure (SBP) in *Fgf21*$^{-/-}$ control mice during daytime. SBP (top panels), SBP when the mouse was walking (moving horizontally, middle panels), and SBP when the mouse was not walking (moving vertically or not moving, bottom panels) were shown (N = 7). The mean and the standard deviation of individual mice were shown in Supplementary Table S4-S6.

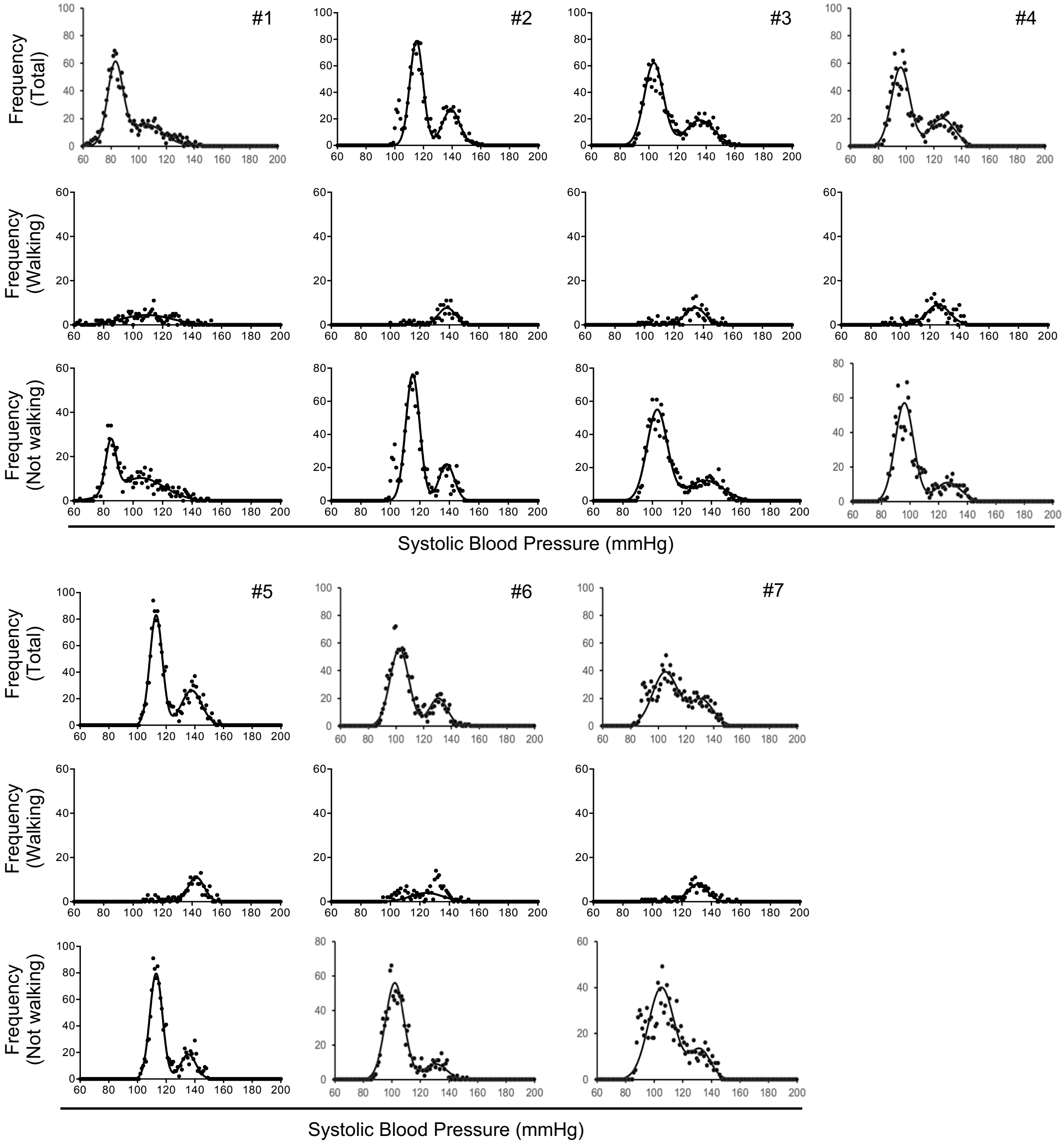

Supplementary Figure S10. Histograms of systolic blood pressure (SBP) in *Fgf21*$^{-/-}$ CKD mice during daytime. SBP (top panels), SBP when the mouse was walking (moving horizontally, middle panels), and SBP when the mouse was not walking (moving vertically or not moving, bottom panels) were shown (N = 7). The mean and the standard deviation of individual mice were shown in Supplementary Table S4-S6.

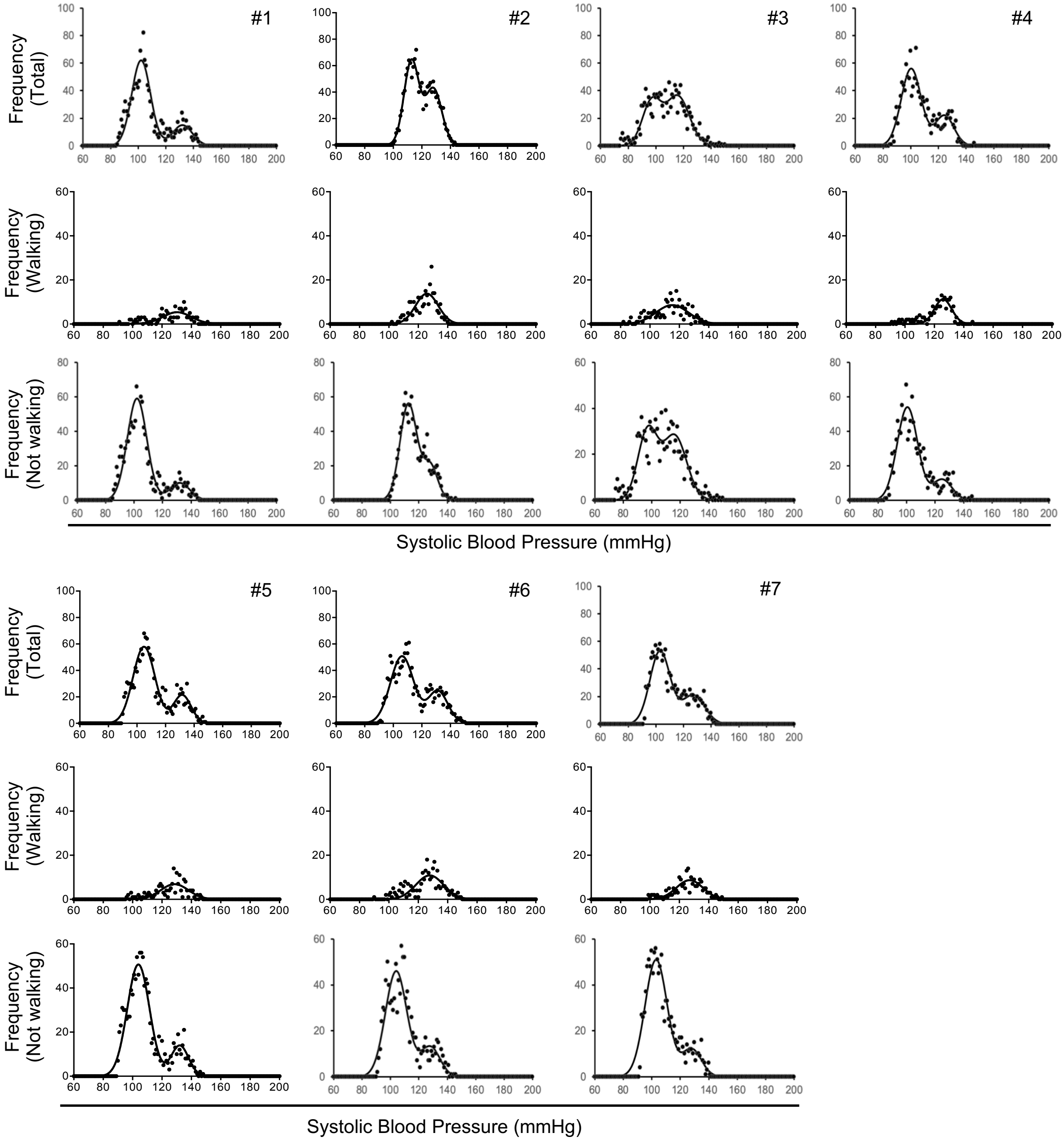

Supplementary Figure S11. Histograms of systolic blood pressure (SBP) in wild-type mice injected with the AAV8-LacZ during daytime. SBP (top panels), SBP when the mouse was walking (moving horizontally, middle panels), and SBP when the mouse was not walking (moving vertically or not moving, bottom panels) were shown (N = 7). The mean and the standard deviation of individual mice were shown in Supplementary Table S4-S6.

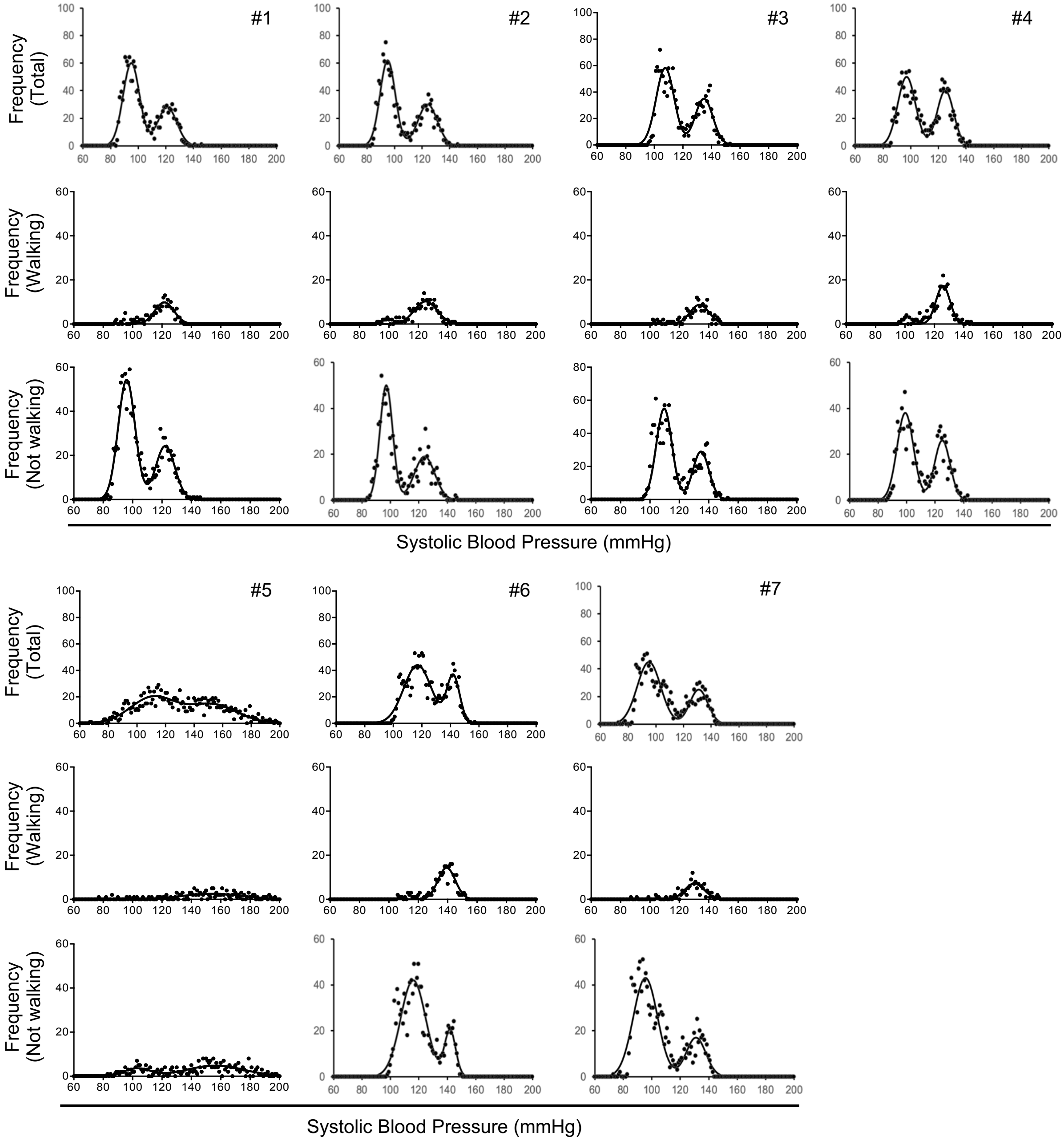

Supplementary Figure S12. Histograms of systolic blood pressure (SBP) in wild-type mice injected with the AAV8-FGF21 during daytime. SBP (top panels), SBP when the mouse was walking (moving horizontally, middle panels), and SBP when the mouse was not walking (moving vertically or not moving, bottom panels) were shown (N = 7). The mean and the standard deviation of individual mice were shown in Supplementary Table S4-S6.

Supplementary Table S1. Mean and standard deviation (SD) of D1 and D2 in the nighttime

Nighttime	D1		D2	
	Mean	SD	Mean	SD
WT Control #1	103.0	7.0	130.0	7.6
WT Control #2	106.0	5.4	122.0	8.0
WT Control #3	96.5	6.0	119.5	5.9
WT Control #4	99.9	5.7	124.0	9.8
WT Control #5	100.8	6.1	128.6	7.8
WT Control #6	102.0	6.1	123.3	7.8
WT Control #7	101.2	5.6	121.0	9.9
Average	101.3	6.0	124.1	8.1

Nighttime	D1		D2	
	Mean	SD	Mean	SD
WT CKD #1	104.5	7.5	134.0	6.5
WT CKD #2	102.0	11.3	129.0	9.1
WT CKD #3	96.5	7.5	132.6	20.4
WT CKD #4	108.6	5.4	143.0	10.3
WT CKD #5	98.3	6.7	131.4	11.6
WT CKD #6	103.8	7.5	136.0	6.9
WT CKD #7	106.7	5.2	130.1	5.8
Average	102.9	7.3	133.7	10.1

Nighttime	D1		D2	
	Mean	SD	Mean	SD
$Fgf21^{-/-}$ Control #1	98.0	7.4	126.5	6.1
$Fgf21^{-/-}$ Control #2	92.0	7.8	125.0	13.0
$Fgf21^{-/-}$ Control #3	127.9	13.2	103.6	6.6
$Fgf21^{-/-}$ Control #4	133.0	9.1	104.8	5.6
$Fgf21^{-/-}$ Control #5	104.0	6.1	123.0	8.1
$Fgf21^{-/-}$ Control #6	100.0	4.9	125.0	6.9
$Fgf21^{-/-}$ Control #7	100.0	4.9	127.0	6.1
Average	107.8	7.6	119.3	7.5

Nighttime	D1		D2	
	Mean	SD	Mean	SD
Fgf21$^{-/-}$ CKD #1	86.5	7.6	113.5	12.0
Fgf21$^{-/-}$ CKD #2	115.1	4.6	139.3	6.1
Fgf21$^{-/-}$ CKD #3	135.7	9.8	103.5	6.6
Fgf21$^{-/-}$ CKD #4	96.0	6.6	126.0	8.9
Fgf21$^{-/-}$ CKD #5	113.0	4.4	137.7	7.1
Fgf21$^{-/-}$ CKD #6	102.5	6.8	130.5	6.5
Fgf21$^{-/-}$ CKD #7	105.0	9.1	131.0	8.4
Average	107.7	7.0	125.9	7.9

Nighttime	D1		D2	
	Mean	SD	Mean	SD
WT AAV8-LacZ #1	102.1	6.8	132.0	7.2
WT AAV8-LacZ #2	112.7	5.2	128.1	5.8
WT AAV8-LacZ #3	97.0	6.8	115.8	8.4
WT AAV8-LacZ #4	100.3	7.2	124.4	6.8
WT AAV8-LacZ #5	104.9	7.7	131.7	6.1
WT AAV8-LacZ #6	106.2	8.1	131.2	6.9
WT AAV8-LacZ #7	103.0	7.3	127.0	8.2
Average	103.7	7.0	127.2	7.1

Nighttime	D1		D2	
	Mean	SD	Mean	SD
WT AAV8-FGF21 #1	95.0	6.0	121.0	6.9
WT AAV8-FGF21 #2	95.0	5.5	124.0	7.0
WT AAV8-FGF21 #3	107.6	6.3	134.8	6.3
WT AAV8-FGF21 #4	97.0	6.4	125.0	6.0
WT AAV8-FGF21 #5	111.2	15.6	152.7	17.3
WT AAV8-FGF21 #6	117.0	9.7	142.0	4.5
WT AAV8-FGF21 #7	95.0	8.5	131.0	6.5
	102.5	8.3	132.9	7.8

Supplementary Table S2. Mean and standard deviation (SD) of D1 and D2 during "walking" in the nighttime

Nighttime	D1		D2	
"Walking"	Mean	SD	Mean	SD
WT Control #1	-	-	135.3	10.1
WT Control #2	-	-	128.5	6.5
WT Control #3	-	-	120.0	6.5
WT Control #4	-	-	128.9	7.3
WT Control #5	-	-	133.4	6.3
WT Control #6	-	-	128.8	5.6
WT Control #7	-	-	125.6	8.2
Average			128.6	7.2

Nighttime	D1		D2	
"Walking"	Mean	SD	Mean	SD
WT CKD #1	-	-	141.8	6.4
WT CKD #2	-	-	136.2	11.2
WT CKD #3	-	-	151.1	8.3
WT CKD #4	-	-	148.3	7.3
WT CKD #5	-	-	142.9	7.7
WT CKD #6	-	-	139.1	5.8
WT CKD #7	-	-	135.0	4.9
Average			142.1	7.4

Nighttime	D1		D2	
"Walking"	Mean	SD	Mean	SD
$Fgf21^{-/-}$ Control #1	-	-	128.0	8.7
$Fgf21^{-/-}$ Control #2	-	-	129.1	9.6
$Fgf21^{-/-}$ Control #3	-	-	132.0	9.0
$Fgf21^{-/-}$ Control #4	-	-	136.2	6.5
$Fgf21^{-/-}$ Control #5	-	-	130.5	6.5
$Fgf21^{-/-}$ Control #6	-	-	127.7	5.5
$Fgf21^{-/-}$ Control #7	-	-	137.0	7.5
Average			131.5	7.6

Nighttime	D1		D2	
"Walking"	Mean	SD	Mean	SD
Fgf21$^{-/-}$ CKD #1	-	-	117.5	15.8
Fgf21$^{-/-}$ CKD #2	-	-	139.3	5.4
Fgf21$^{-/-}$ CKD #3	-	-	139.4	4.9
Fgf21$^{-/-}$ CKD #4	-	-	133.2	8.3
Fgf21$^{-/-}$ CKD #5	-	-	116.2	13.9
Fgf21$^{-/-}$ CKD #6	-	-	124.6	7.7
Fgf21$^{-/-}$ CKD #7	-	-	136.8	5.3
Average			129.6	8.8

Nighttime	D1		D2	
"Walking"	Mean	SD	Mean	SD
WT AAV8-LacZ #1	-	-	135.5	11.3
WT AAV8-LacZ #2	-	-	127.3	5.8
WT AAV8-LacZ #3	-	-	121.3	8.8
WT AAV8-LacZ #4	-	-	126.1	8.3
WT AAV8-LacZ #5	-	-	132.9	7.0
WT AAV8-LacZ #6	-	-	131.3	6.1
WT AAV8-LacZ #7	-	-	128.0	8.5
Average			128.9	8.0

Nighttime	D1		D2	
"Walking"	Mean	SD	Mean	SD
WT AAV8-FGF21 #1	-	-	130.8	5.8
WT AAV8-FGF21 #2	-	-	133.0	7.6
WT AAV8-FGF21 #3	-	-	144.2	5.3
WT AAV8-FGF21 #4	-	-	133.2	7.8
WT AAV8-FGF21 #5	-	-	136.0	8.1
WT AAV8-FGF21 #6	-	-	138.6	9.4
WT AAV8-FGF21 #7	-	-	131.1	6.5
			135.3	7.2

Supplementary Table S3. Mean and standard deviation (SD) of D1 and D2 during "not walking" in the nighttime

Nighttime	D1		D2	
"Not walking"	Mean	SD	Mean	SD
WT Control #1	106.2	5.7	128.7	7.6
WT Control #2	109.0	7.1	124.5	7.5
WT Control #3	110.0	8.5	122.0	6.9
WT Control #4	101.4	6.7	125.0	7.8
WT Control #5	107.4	6.2	128.9	6.4
WT Control #6	106.0	6.8	129.4	6.1
WT Control #7	101.4	1.8	121.3	9.2
Average	105.9	6.1	125.7	7.3

Nighttime	D1		D2	
"Not walking"	Mean	SD	Mean	SD
WT CKD #1	103.6	5.5	139.0	5.7
WT CKD #2	111.5	9.2	134.9	11.2
WT CKD #3	103.0	12.7	155.1	9.1
WT CKD #4	134.6	18.3	152.2	3.7
WT CKD #5	108.1	6.1	143.7	7.6
WT CKD #6	112.4	8.9	138.9	5.6
WT CKD #7	107.8	4.8	132.5	5.4
Average	111.6	9.3	142.3	6.9

Nighttime	D1		D2	
"Not walking"	Mean	SD	Mean	SD
$Fgf21^{-/-}$ Control #1	108.0	5.1	128.3	6.3
$Fgf21^{-/-}$ Control #2	99.0	5.2	119.0	11.8
$Fgf21^{-/-}$ Control #3	109.0	5.3	127.0	9.5
$Fgf21^{-/-}$ Control #4	110.0	6.0	134.0	8.7
$Fgf21^{-/-}$ Control #5	115.0	3.9	126.0	6.9
$Fgf21^{-/-}$ Control #6	106.1	4.4	126.7	5.7
$Fgf21^{-/-}$ Control #7	109.7	5.2	130.8	6.7
Average	108.1	5.0	127.4	7.9

Nighttime	D1		D2	
“Not walking”	Mean	SD	Mean	SD
$Fgf21^{-/-}$ CKD #1	93.0	9.1	119.0	10.0
$Fgf21^{-/-}$ CKD #2	104.0	5.2	130.0	9.0
$Fgf21^{-/-}$ CKD #3	116.3	5.1	138.8	8.0
$Fgf21^{-/-}$ CKD #4	105.9	6.6	137.4	10.1
$Fgf21^{-/-}$ CKD #5	95.0	5.0	116.5	8.3
$Fgf21^{-/-}$ CKD #6	102.0	6.9	123.0	6.9
$Fgf21^{-/-}$ CKD #7	107.0	6.3	134.6	5.8
Average	103.3	6.3	128.5	8.3

Nighttime	D1		D2	
“Not walking”	Mean	SD	Mean	SD
WT AAV8-LacZ #1	107.5	5.6	129.3	7.1
WT AAV8-LacZ #2	109.0	5.8	126.6	6.1
WT AAV8-LacZ #3	119.0	8.0	147.3	4.2
WT AAV8-LacZ #4	105.0	6.5	122.5	9.0
WT AAV8-LacZ #5	113.3	9.8	133.7	5.6
WT AAV8-LacZ #6	109.4	6.4	129.0	7.1
WT AAV8-LacZ #7	109.2	6.8	128.3	6.3
Average	110.3	7.0	131.0	6.5

Nighttime	D1		D2	
“Not walking”	Mean	SD	Mean	SD
WT AAV8-FGF21 #1	106.8	6.3	130.3	7.1
WT AAV8-FGF21 #2	116.7	5.6	135.2	7.4
WT AAV8-FGF21 #3	119.4	7.0	144.2	5.3
WT AAV8-FGF21 #4	109.1	7.0	132.6	8.8
WT AAV8-FGF21 #5	108.8	8.6	136.7	6.1
WT AAV8-FGF21 #6	117.5	8.0	141.8	7.3
WT AAV8-FGF21 #7	92.0	3.2	121.8	8.0
Average	110.0	6.5	134.7	7.2

Supplementary Table S4. Mean and standard deviation (SD) of D1 and D2 in the Daytime

Daytime	D1		D2	
	Mean	SD	Mean	SD
WT Control #1	103.0	7.0	130.0	7.6
WT Control #2	106.0	5.4	122.0	8.0
WT Control #3	96.5	6.0	119.5	5.9
WT Control #4	99.9	5.7	124.0	9.8
WT Control #5	100.8	6.1	128.6	7.8
WT Control #6	102.0	6.1	123.3	7.8
WT Control #7	101.2	5.6	121.0	9.9
Average	101.3	6.0	124.1	8.1

Daytime	D1		D2	
	Mean	SD	Mean	SD
WT CKD #1	104.5	7.5	134.0	6.5
WT CKD #2	102.0	11.3	129.0	9.1
WT CKD #3	96.5	7.5	132.6	20.4
WT CKD #4	108.6	5.4	143.0	10.3
WT CKD #5	98.3	6.7	131.4	11.6
WT CKD #6	103.8	7.5	136.0	6.9
WT CKD #7	106.7	5.2	130.1	5.8
Average	102.9	7.3	133.7	10.1

Daytime	D1		D2	
	Mean	SD	Mean	SD
$Fgf21^{-/-}$ Control #1	98.0	7.4	126.5	6.1
$Fgf21^{-/-}$ Control #2	92.0	7.8	125.0	13.0
$Fgf21^{-/-}$ Control #3	127.9	13.2	103.6	6.6
$Fgf21^{-/-}$ Control #4	133.0	9.1	104.8	5.6
$Fgf21^{-/-}$ Control #5	104.0	6.1	123.0	8.1
$Fgf21^{-/-}$ Control #6	100.0	4.9	125.0	6.9
$Fgf21^{-/-}$ Control #7	100.0	4.9	127.0	6.1
Average	107.8	7.6	119.3	7.5

Daytime	D1		D2	
	Mean	SD	Mean	SD
$Fgf21^{-/-}$ CKD #1	86.5	7.6	113.5	12.0
$Fgf21^{-/-}$ CKD #2	115.1	4.6	139.3	6.1
$Fgf21^{-/-}$ CKD #3	135.7	9.8	103.5	6.6
$Fgf21^{-/-}$ CKD #4	96.0	6.6	126.0	8.9
$Fgf21^{-/-}$ CKD #5	113.0	4.4	137.7	7.1
$Fgf21^{-/-}$ CKD #6	102.5	6.8	130.5	6.5
$Fgf21^{-/-}$ CKD #7	105.0	9.1	131.0	8.4
Average	107.7	7.0	125.9	7.9

Daytime	D1		D2	
	Mean	SD	Mean	SD
WT AAV8-LacZ #1	102.1	6.8	132.0	7.2
WT AAV8-LacZ #2	112.7	5.2	128.1	5.8
WT AAV8-LacZ #3	97.0	6.8	115.8	8.4
WT AAV8-LacZ #4	100.3	7.2	124.4	6.8
WT AAV8-LacZ #5	104.9	7.7	131.7	6.1
WT AAV8-LacZ #6	106.2	8.1	131.2	6.9
WT AAV8-LacZ #7	103.0	7.3	127.0	8.2
Average	103.7	7.0	127.2	7.1

Daytime	D1		D2	
	Mean	SD	Mean	SD
WT AAV8-FGF21 #1	95.0	6.0	121.0	6.9
WT AAV8-FGF21 #2	95.0	5.5	124.0	7.0
WT AAV8-FGF21 #3	107.6	6.3	134.8	6.3
WT AAV8-FGF21 #4	97.0	6.4	125.0	6.0
WT AAV8-FGF21 #5	111.2	15.6	152.7	17.3
WT AAV8-FGF21 #6	117.0	9.7	142.0	4.5
WT AAV8-FGF21 #7	95.0	8.5	131.0	6.5
Average	102.5	8.3	132.9	7.8

Supplementary Table S5. Mean and standard deviation (SD) of D1 and D2 during "walking" in the daytime

Daytime	D1		D2	
"Walking"	Mean	SD	Mean	SD
WT Control #1	-	-	124.7	11.9
WT Control #2	-	-	121.8	7.1
WT Control #3	-	-	117.6	7.3
WT Control #4	-	-	126.7	5.3
WT Control #5	-	-	127.9	8.2
WT Control #6	-	-	125.1	9.0
WT Control #7	-	-	123.3	13.2
Average			123.9	8.8

Daytime	D1		D2	
"Walking"	Mean	SD	Mean	SD
WT CKD #1	-	-	134.4	7.3
WT CKD #2	-	-	119.6	15.3
WT CKD #3	-	-	141.1	11.8
WT CKD #4	-	-	143.6	5.6
WT CKD #5	-	-	134.3	8.1
WT CKD #6	-	-	136.8	8.3
WT CKD #7	-	-	131.0	6.8
Average			134.4	9.0

Daytime	D1		D2	
"Walking"	Mean	SD	Mean	SD
$Fgf21^{-/-}$ Control #1	-	-	127.0	7.6
$Fgf21^{-/-}$ Control #2	-	-	125.9	12.4
$Fgf21^{-/-}$ Control #3	-	-	127.7	13.5
$Fgf21^{-/-}$ Control #4	-	-	134.0	6.4
$Fgf21^{-/-}$ Control #5	-	-	126.6	7.8
$Fgf21^{-/-}$ Control #6	-	-	125.4	7.4
$Fgf21^{-/-}$ Control #7	-	-	129.2	5.6
Average			128.0	8.7

Daytime	D1		D2	
“Walking”	Mean	SD	Mean	SD
Fgf21$^{-/-}$ CKD #1	-	-	109.2	17.0
Fgf21$^{-/-}$ CKD #2	-	-	138.4	5.8
Fgf21$^{-/-}$ CKD #3	-	-	134.4	6.7
Fgf21$^{-/-}$ CKD #4	-	-	125.7	7.7
Fgf21$^{-/-}$ CKD #5	-	-	142.8	5.5
Fgf21$^{-/-}$ CKD #6	-	-	124.6	11.0
Fgf21$^{-/-}$ CKD #7	-	-	130.7	6.7
Average			129.4	8.6

Daytime	D1		D2	
“Walking”	Mean	SD	Mean	SD
WT AAV8-LacZ #1	-	-	130.5	8.0
WT AAV8-LacZ #2	-	-	125.9	7.7
WT AAV8-LacZ #3	-	-	115.2	11.1
WT AAV8-LacZ #4	-	-	125.9	5.9
WT AAV8-LacZ #5	-	-	128.6	9.4
WT AAV8-LacZ #6	-	-	127.8	9.5
WT AAV8-LacZ #7	-	-	127.0	9.0
Average			125.8	8.7

Daytime	D1		D2	
“Walking”	Mean	SD	Mean	SD
WT AAV8-FGF21 #1	-	-	121.6	6.6
WT AAV8-FGF21 #2	-	-	125.1	7.7
WT AAV8-FGF21 #3	-	-	133.8	6.5
WT AAV8-FGF21 #4	-	-	125.9	4.8
WT AAV8-FGF21 #5	-	-	154.6	20.9
WT AAV8-FGF21 #6	-	-	139.3	5.9
WT AAV8-FGF21 #7	-	-	130.3	6.5
Average			132.9	8.4

Supplementary Table S6. Mean and standard deviation (SD) of D1 and D2 during "not walking" in the daytime

Daytime	D1		D2	
"Not walking"	Mean	SD	Mean	SD
WT Control #1	103.1	6.4	127.6	11.1
WT Control #2	107.0	6.6	127.0	6.3
WT Control #3	96.8	5.7	119.9	6.0
WT Control #4	99.6	6.3	123.3	9.2
WT Control #5	100.3	6.1	128.6	8.1
WT Control #6	102.0	6.1	123.3	8.1
WT Control #7	100.2	5.7	122.0	9.9
Average	101.3	6.1	124.5	8.4

Daytime	D1		D2	
"Not walking"	Mean	SD	Mean	SD
WT CKD #1	103.2	7.4	132.4	7.6
WT CKD #2	105.4	11.6	132.0	8.4
WT CKD #3	105.0	11.2	142.3	13.5
WT CKD #4	106.8	4.1	135.4	18.6
WT CKD #5	97.5	7.0	133.5	8.0
WT CKD #6	104.5	7.2	136.0	6.8
WT CKD #7	106.7	4.9	129.5	5.5
Average	104.2	7.6	134.4	9.8

Daytime	D1		D2	
"Not walking"	Mean	SD	Mean	SD
$Fgf21^{-/-}$ Control #1	98.0	7.4	126.0	5.8
$Fgf21^{-/-}$ Control #2	92.0	7.4	123.0	13.0
$Fgf21^{-/-}$ Control #3	103.0	6.9	120.0	16.0
$Fgf21^{-/-}$ Control #4	104.0	5.5	133.0	9.2
$Fgf21^{-/-}$ Control #5	104.0	6.5	123.0	7.2
$Fgf21^{-/-}$ Control #6	99.0	4.7	125.0	6.8
$Fgf21^{-/-}$ Control #7	99.5	4.7	125.0	5.9
Average	99.9	6.2	125.0	9.1

Daytime	D1		D2	
"Not walking"	Mean	SD	Mean	SD
Fgf21$^{-/-}$ CKD #1	85.0	3.6	105.1	16.1
Fgf21$^{-/-}$ CKD #2	115.2	4.7	138.2	5.1
Fgf21$^{-/-}$ CKD #3	103.2	6.9	135.7	12.3
Fgf21$^{-/-}$ CKD #4	96.0	6.5	126.5	8.9
Fgf21$^{-/-}$ CKD #5	113.0	4.4	135.5	5.4
Fgf21$^{-/-}$ CKD #6	102.0	6.6	130.5	7.8
Fgf21$^{-/-}$ CKD #7	105.0	9.1	132.0	7.2
Average	102.8	6.0	129.1	9.0

Daytime	D1		D2	
"Not walking"	Mean	SD	Mean	SD
WT AAV8-LacZ #1	102.0	6.9	132.0	7.1
WT AAV8-LacZ #2	112.7	6.0	128.0	5.2
WT AAV8-LacZ #3	97.0	6.8	116.0	8.4
WT AAV8-LacZ #4	100.5	7.2	125.0	6.8
WT AAV8-LacZ #5	104.0	7.6	132.1	5.9
WT AAV8-LacZ #6	104.0	7.6	128.0	6.8
WT AAV8-LacZ #7	103.0	7.8	128.0	6.8
Average	103.3	7.1	127.0	6.7

Daytime	D1		D2	
"Not walking"	Mean	SD	Mean	SD
WT AAV8-FGF21 #1	96.0	5.8	122.3	6.5
WT AAV8-FGF21 #2	95.0	5.5	124.0	7.0
WT AAV8-FGF21 #3	107.6	6.3	134.8	6.3
WT AAV8-FGF21 #4	97.0	6.4	125.0	6.0
WT AAV8-FGF21 #5	111.2	15.6	152.7	17.3
WT AAV8-FGF21 #6	117.0	9.7	142.0	4.5
WT AAV8-FGF21 #7	95.0	8.5	131.0	6.5
Average	103.3	7.1	127.0	6.7

Supplementary Table S7. Characteristics of selected participants (n =185)

Variables	Total		
Age (years)	65	±	8
Women, n (%)	108 (58)		
Body mass index (kg/m^2)	22.9	±	3.4
Visceral fat (cm^2)*	60	±	31
eGFR (mL/min/1.73m^2)	67	±	20
Systolic blood pressure (mmHg)	125	±	16
Diastolic blood pressure (mmHg)	76	±	10
Carotid-femoral PWV (cm/sec)	992	±	213
Baroreflex sensitivity (ms/mmHg)	5.3	±	3.0
Serum FGF21 levels (pg/mL)	135	±	148
Anti-hypertensive medication, n (%)	79 (43)		
Current smoking status, n (%)	3 (2)		

Data are presented as the means ± SD or frequency counts (%). eGFR, average value of estimated glomerular filtration rate calculated from serum creatinine or cystatin C; PWV, pulse wave velocity; FGF21, fibroblast growth factor-21. **N* = 184.

Supplementary Table S8. Independent determinates of baroreflex sensitivity

Variables	B	±	SE	β	*P*
**R^2 =0.144**					
Gender (woman)	-0.157	±	0.035	-0.318	< 0.001
Age (years)	-0.003	±	0.002	-0.113	0.130
Serum FGF21 levels (pg/mL)*	-0.082	±	0.050	-0.120	0.100
Carotid-femoral PWV (cm/sec)*	-0.441	±	0.218	-0.157	0.044

Covariates in the stepwise linear regression model included age, gender (woman), body mass index, visceral fat (*N* = 184), eGFR, current smoking status, anti-hypertensive medication, and serum FGF21 levels. Abbreviations: eGFR, average of estimated glomerular filtration rate calculated from serum creatinine and that from cystatin C; PWV, pulse wave velocity. *Log-transformed.

Supplementary Table S9. Primers used for quantitative RT-PCR

	Forward	Reverse
Osteopontin	TCCAAAGAGAGCCAGGAGAG	GGCTTTGGAACTTGCTTGAC
Ngal	GAAATATGCACAGGTATCCTC	GTAATTTTGAAGTATTGCTTGTTT
MCP1	GGCTCAGCCAGATGCAGTTAAC	GCCTACTCATTGGGATCATCTTG
Runx2	TCTGGGAGGAAGGAGAGACA	GACTCTGTAAGCGGGTCTGC
Osteocalcin	TGCCCTAAAGCCAAACTCTG	ACTTGCAGGGCAGAGAGAGA
Cyclophilin	TGGAGAGCACCAAGACAGACA	TGCCGGAGTCGACAATGAT
